# Correction to: High-quality genome assemblies from key Hawaiian coral species

**DOI:** 10.1093/gigascience/giad027

**Published:** 2023-04-11

This is a correction to: Timothy G Stephens, JunMo Lee, YuJin Jeong, Hwan Su Yoon, Hollie M Putnam, Eva Majerová, Debashish Bhattacharya, High-quality genome assemblies from key Hawaiian coral species, *GigaScience*, Volume 11,2022, giac098, https://doi.org/10.1093/gigascience/giac098

In the originally published version of this manuscript, there was an error in the title. This should read: “High-quality genome assemblies from key Hawaiian coral species” instead of “High-quality genome assembles from key Hawaiian coral species.”

This error has been corrected in the article.

