# Supplementary material for: High-quality genome assembles from key Hawaiian coral species
Source: Gigascience. 2022 Nov 9;11:giac098. doi: 10.1093/gigascience/giac098 (PMC9646523; doi:10.1093/gigascience/giac098)
Supplement: giac098_GIGA-D-22-00143_Revision_1 [file giac098_giga-d-22-00143_revision_1.pdf]

GigaScience

High-quality genome assembles from key Hawaiian coral species

--Manuscript Draft--

**Manuscript Number:**	GIGA-D-22-00143R1
**Full Title:**	High-quality genome assembles from key Hawaiian coral species
**Article Type:**	Data Note

Funding Information:

USDA National Institute of Food and Agriculture (1017848)	Dr Hollie M. Putnam
National Science Foundation (NSF-OCE 1756623)	Dr Hollie M. Putnam
National Science Foundation (NSF-OCE 1756616)	Dr Debashish Bhattacharya
Catalyst Science Fund (2020-008)	Dr Debashish Bhattacharya
National Institute of Food and Agriculture and United States Department of Agriculture (NJ01180)	Dr Debashish Bhattacharya
National Aeronautics and Space Administration (80NSSC19K0462)	Dr Debashish Bhattacharya
Ministry of Oceans and Fisheries (20180430)	Dr Hwan Su Yoon
National Research Foundation of Korea (2020R1C1C1010193)	Dr JunMo Lee
Korea Ministry of Environment (2021003420004)	Dr JunMo Lee
Paul G. Allen Family Foundation	Dr Eva Majerová

Abstract:

Background

Coral reefs house about 25% of marine biodiversity and are critical for the livelihood of many communities by providing food, tourism revenue, and protection from wave surge. These magnificent ecosystems are under existential threat from anthropogenic climate change. Whereas extensive ecological and physiological studies have addressed coral response to environmental stress, high-quality reference genome data are lacking for many of these species. The latter issue hinders efforts to understand the genetic basis of stress resistance and to design informed coral conservation strategies.

Results

We report genome assemblies from four key Hawaiian coral species, Montipora capitata , Pocillopora acuta , Pocillopora meandrina , and Porites compressa . These species, or members of these genera, are distributed worldwide and therefore of broad scientific and ecological importance. For M. capitata , an initial assembly was generated from short-read Illumina and long-read PacBio data, which was then scaffolded into 14 putative chromosomes using Omni-C sequencing. For Poc. acuta , Poc. meandrina , and Por. compressa , high-quality assemblies were generated using short-read Illumina and long-read PacBio data. The Poc. acuta assembly is from a triploid individual, making it the first reference genome of a non-diploid coral animal.

Conclusions

These assemblies are significant improvements over available data and provide invaluable resources for supporting multi-omics studies into coral biology, not just in Hawai‘i, but also in other regions, where related species exist. The Poc. acuta assembly provides a platform for studying polyploidy in corals and its role in genome evolution and stress adaptation in these organisms.

**Corresponding Author:**	Timothy Gordon Stephens, Ph.D.<br>Rutgers University: Rutgers The State University of New Jersey<br>New Brunswick, New Jersey UNITED STATES
**Corresponding Author Secondary**	

Powered by Editorial Manager® and ProduXion Manager® from Aries Systems Corporation

**Information:**	
**Corresponding Author's Institution:**	Rutgers University: Rutgers The State University of New Jersey
**Corresponding Author's Secondary Institution:**	
**First Author:**	Timothy Gordon Stephens, Ph.D.
**First Author Secondary Information:**	
**Order of Authors:**	Timothy Gordon Stephens, Ph.D.
	JunMo Lee
	YuJin Jeong
	Hwan Su Yoon
	Hollie M. Putnam
	Eva Majerová
	Debashish Bhattacharya
**Order of Authors Secondary Information:**	
**Response to Reviewers:**	Dear Editor,<br>We thank the two reviewers for their constructive comments on our earlier manuscript. In this submission, we revised our manuscript based on all of these comments, and to improve readability. We have added two new supplementary tables listing the SRA Run IDs and results of functional annotation, and a figure showing our assembly and gene prediction workflow. We also added a more detailed description of the symbiont filtering approach and the results of functional annotation.<br><br>Reviewer #1: Stephens et al. reported de novo genome assemblies from four coral species in Hawaii. They constructed a chromosome-level assembly of Montipora capitata using the Omni-C sequencing technology. These genome assemblies surpass previous ones from the same species or genera in contiguity and BUSCO completeness. These genome assemblies will be helpful to the coral research community. I have a few comments for the authors to consider.<br><br>The authors would benefit from proof-read by an English editor to correct grammar and improve the manuscript's readability.<br><br>We have extensively reviewed the grammar and phrasing of the manuscript to improve its readability.<br><br>Lines 139-151, 182-191<br>I think it is better to summarize the information of the sequence data in tables than to describe it in the text.<br><br>We have added a new supplementary table (Table S1) listing the IDs of the SRA Runs used for genome assembly and gene prediction in this study. We have removed the lists of Run IDs from the main text and now refer to the new table where appropriate.<br><br>L144-154: “The PacBio reads from M. capitata (78.3 Gbp; Supplementary Table S1) and Por. compressa (63.3 Gbp) were generated using the PacBio RSII platform (giving the ‘-pacbio’ parameter to the CANU assembler). The PacBio reads for Poc. meandrina (311.8 Gbp; Supplementary Table S1), and Poc. acuta (239.1 Gbp) were generated using the PacBio HiFi platform (giving the ‘-pacbio-hifi’ parameter to the CANU assembler). An error correction step (nucleotide correction of assembly) using the initial assemblies of M. capitata (1.2 Gbp; Supplementary Table S2), Por. compressa (1.0 Gbp), Poc. meandrina (0.7 Gbp), and Poc. acuta (1.1 Gbp) was done using bowtie2 (v2.4.2; default options) [31] and the Pilon program (v1.23; default options) [28] with the Illumina short-read sequencing data (27.4 Gbp for M. capitata; 20.9 Gbp for Por. compressa; 27.2 Gbp for Poc. meandrina, and 23.0 Gbp for Poc. acuta; Supplementary Table S1).”

L202-205: “Quality trimming and adapter removal from the RNA sequencing (RNA-seq) data in the Hawaiian coral species (77.5 Gbp for M. capitata, 76.5 Gbp for Por. compressa, 656.7 Gbp for Poc. acuta, and 10.6 Gbp for Poc. meandrina; Supplementary Table S1) were done using Trimmomatic (v0.39; default options) [29].”

L527-529: “The SRA Run IDs of the Omni-C data generated from the Hawaiian M. capitata, the PacBio and Illumina genome data used for genome assembly, and the RNA-seq data used for gene prediction are listed in Supplementary Table S1 for each species.”

Lines 203-205
Results of functional annotation are not described.

We had added to the manuscript additional text describing these results and a new supplementary table (Table S8) that lists the number of functionally annotated genes in each species.

L422-424: “In the new assembly, 56.68% of the predicted protein-coding genes were assigned putative functions using CD-Search, 44.26% using eggNOG-mapper, and 21.20% using KAAS (Supplementary Table S8).”

L442-446: “In Poc. acuta, 67.76% of the predicted protein-coding genes were assigned putative functions using CD-Search, 49.76% using eggNOG-mapper, and 32.35% using KAAS, and in Poc. meandrina, 69.44% of the predicted protein-coding genes were assigned putative functions using CD-Search, 51.76% using eggNOG-mapper, and 33.66% using KAAS (Supplementary Table S8).”

L469-471: “In Por. compressa, 63.91% of the predicted protein-coding genes were assigned putative functions using CD-Search, 46.22% using eggNOG-mapper, and 27.48% using KAAS (Supplementary Table S8).”

L783-784: “Table S8: Number of predicted protein-coding genes in each of the new Hawaiian coral genomes with functional annotations.”

Reviewer #2: n this work, Stephens et al present improved reference genomes from four Hawaian coral species using a combination of short and long read sequencing as well as linkage information in one assembly. They also sequence the first triploid coral. I believe this data will be a valuable resource to the larger coral community and are thus a good fit for a GigaScience Data Note. Overall, the methods are largely sound, appropriate and reproducible. Some small suggestions to improve are:

1) The manuscript would benefit from workflow diagrams describing the entire workflow and potentially a separate diagram for the assembly and annotation pipeline.

We agree with the reviewer and have added a diagram of the genome assembly, gene prediction, and functional annotation workflow to the manuscript.

L141-142: “A diagram depicting the genome assembly, gene prediction, and functional annotation workflow used for each of the Hawaiian coral species is presented in Figure 1.”

L787-790: “Figure 1: Diagram depicting the genome assembly, gene prediction, and functional annotation workflow deployed in this study to assemble each of the new Hawaiian coral genomes. Programs are presented in green boxes and datasets in dark orange boxes, arrows show the flow of data through the workflow. Major input and output datasets are highlighted with bold text.”

2) The improved assemblies will be beneficial to the research community. Could you clarify whether the old assemblies were utilised in any way during the construction of the improved assemblies?

We thank the Reviewer for their support of the importance of these data to the research community. The old assemblies were not used in any way during the

Powered by Editorial Manager® and ProduXion Manager® from Aries Systems Corporation

construction of the improved assemblies. As we describe in the methods, the “long-read genome sequencing data (PacBio) of the Hawaiian coral species were initially assembled using CANU (v2.2; default options)”. That is, each of the improved assemblies were constructed directly from the long and short read data and not using the existing genome assemblies as a start point. As we feel that this is adequately described in the manuscript, we have made no further changes.

3) L204: "Functional annotation of gene models was done using the NCBI Conserved Domain Search (CD-Search) [42], the eggNOG-mapper [43], and the KEGG Automatic Annotation Server (KAAS)". Is this functional data described in the manuscript? Is it available?

We will be making the results of functional annotation available through our lab website and the GigaDB data repository. We have also added to the manuscript additional text describing the functional annotation results, as well as a new supplementary table (Table S8) that lists the number of functionally annotated genes in each species.

L529-535: “The genome assemblies, predicted genes, and functional annotations for the Hawaiian M. capitata is available from http://cyanophora.rutgers.edu/montipora/ (Version 3), for Poc. acuta from http://cyanophora.rutgers.edu/Pocillopora_acuta/ (Version 2), Poc. meandrina from http://cyanophora.rutgers.edu/Pocillopora_meandrina/ (Version 1), Por. compressa from http://cyanophora.rutgers.edu/Porites_compressa/ (Version 1). The data associated with this manuscript are also available from GigaDB.”

L422-424: “In the new assembly, 56.68% of the predicted protein-coding genes were assigned putative functions using CD-Search, 44.26% using eggNOG-mapper, and 21.20% using KAAS (Supplementary Table S8).”

L442-446: “In Poc. acuta, 67.76% of the predicted protein-coding genes were assigned putative functions using CD-Search, 49.76% using eggNOG-mapper, and 32.35% using KAAS, and in Poc. meandrina, 69.44% of the predicted protein-coding genes were assigned putative functions using CD-Search, 51.76% using eggNOG-mapper, and 33.66% using KAAS (Supplementary Table S8).”

L469-471: “In Por. compressa, 63.91% of the predicted protein-coding genes were assigned putative functions using CD-Search, 46.22% using eggNOG-mapper, and 27.48% using KAAS (Supplementary Table S8).”

L783-784: “Table S8: Number of predicted protein-coding genes in each of the new Hawaiian coral genomes with functional annotations.”

4) You note large differences in the number of predicted genes between species and mention assemblies qualities may impact this. Was there anything characteristic about the genes found uniquely in Por. Compress versus the other assemblies? Did you examine whether there are any functional differences between the genes?

We thank the reviewer for their insightful comment and agree that an exploration of the genes that are unique to the Por. compressa genome would make for an interesting follow-up study. We however think that such an analysis is outside the scope of a GigaScience Data Note article because it would require extensive reanalysis of the published Porites genomes (to ensure the conclusions drawn from the analysis are not the result of differences in assembly and gene prediction quality or methodology) and the exploration and discussion of the literature on Porites and coral genome evolution. We are currently performing follow-up analyses of the genomes that we are publishing in this study, plus all published coral genomes, to explore how the different forces that have shaped the genome evolution of different coral groups. As such, we believe that a rigorous analysis of the genes that are unique to the Por. compressa genome is outside the scope of a GigaScience Data Note article and we have made no additional changes to the manuscript.

5) You state "the best (longest) gene models were manually selected based on results of BLASTp search" however this is not always true. For the two methods, do you have the breakdown for the number of times the transcripts differed and if so which method

predicted the longer transcript?

When gene models from the two types of gene prediction approached are visualized, using for example Geneious Prime, the differently predicted gene models are easily recognized. 'The best (longest) gene models' means that the "best" gene models from the two prediction approaches were selected based on a web-BLASTp search and selection of the longest non-chimeric gene models. We agree with the Reviewer that a BLASTp search will not always return the "true" gene model, however, we propose that a gene model with multiple BLASTp hits to proteins in an updated reference database should be regarded as the strongest evidence of the correct gene structure in the absence of other evidence. To select the longest non-chimeric gene models, we compared gene models (not transcripts) constructed by BRAKER using assembled transcripts or RNA-seq reads as evidence for exons. Further, both type of gene models were used because assembled transcriptome data could generally (but not always) make longer gene models, however, it can also sometimes result in chimeric gene models when UTR regions of two closely related genes overlap. There for, we used gene models from these two complementary methods, and evidence of potential chimeric gene models based on the blast results compared to reference proteins, as the basis for our selection of the "best" non-chimeric gene models. We have rephased this section of the manuscript to make this point clearer. We did not keep track of the number of differently predicted gene models or the number of times one type of prediction was correct over the other.

L213-217: "When the gene models predicted in the same region of the genome by the two gene prediction approaches (i.e., RNA-seq and assembled transcript-based BRAKER gene models) differed, the best (e.g., longest non-chimeric) gene model was manually selected, based on the results of a web-BLASTp search (e-value cutoff = 1.e-5 cutoff)."

6) Could you further explain how symbiont sequence data was handled? For one species you say "from a colony that was greatly reduced in algal symbionts" but for others no such claims are made. You speak of general contamination filtering strategies but given this is coral you might want to specifically describe if anything specific was done for the handling of symbiont sequence.

For M. capitata, Poc. acuta, and Poc. meandrina, DNA was extracted from bleached coral nubbins, which would have reduced algal symbiont densities, and for Por. compressa, DNA was extracted from sperm, which should be free from algal symbionts. As the reviewer highlighted, this is described in the methods for M. capitata and Por. compressa but not for Poc. acuta, and Poc. meandrina. We have added these missing details to the methods section of the manuscript.

L92-93 & 104-105: "This nubbin was selected for DNA extraction as it was bleached and would have a greatly reduced algal symbiont density."

We have added a detailed description of the symbiont sequence screening workflow to the main text of the manuscript; two additional supplementary tables were added that describe the symbiont genome assemblies used for screening and the putative functions of the coral scaffolds identified as having similarity to symbiont genomes above our chosen thresholds.

L160-176: "An additional step was performed to identify any scaffolds in the coral genome assemblies that are putatively derived from the algal (Symbiodiniaceae) symbionts. Each of the four assemblies was compared against a custom database of all published Symbiodiniaceae genomes [23, 31-35] (Supplementary Table S3) using BLASTn (v2.10.1; -max_target_seqs 2000). The resulting BLAST hits were filtered, retaining only those with an e-value < 1e-20 and a bitscore > 1000. Hits to the Cladocopium sp. C15 genome [23] were also removed because this assembly is from a holobiont sequencing project (i.e., was assembled from a metagenome sample) and is, therefore, more likely to be contaminated with coral sequences than the other Symbiodiniaceae data that were derived from unialgal cultures. Overlapping filtered BLAST hits were merged and their coverage of each coral scaffold was calculated using bedtools (v2.29.2) [36]. The regions covered by merged BLAST hits on scaffolds with >10% and >1% of their bases covered by BLASTn hits were extracted and

	compared against the NCBI nt database using the online BLASTn tool (default settings; accessed 21 July 2022). All of the regions on scaffolds with >10% and >1% hit coverage had similarity to coral rRNA sequences in the NCBI nt database (Supplementary Table S4), suggesting that their similarity to Symbiodiniaceae genomes does not represent contamination. Therefore, no additional scaffolds were removed from the coral genome assemblies."<br><br>L767-771: "Table S3: List of Symbiodiniaceae genomes used to assess symbiont contamination in the coral genome assemblies.<br><br>Table S4: Top 10 BLASTn hits against the NCBI's nt database for regions of coral scaffolds with greater than a given coverage of hits to Symbiodiniaceae assembled genomes."<br><br>7) In Figure 1A/B, it would be clearer to highlight the region blown up in the magnified images.<br><br>We agree with the Reviewer that highlighting the magnified regions would make Figure 1A and 1B (now Figure 2) clearer. We have added green bars to each of the panels to highlight the magnified regions.<br><br>L795-798: "In (A) and (B) a zoomed-in section of the larger plot (indicated by a green bar along the x-axis) is shown on the right highlighting the 40 largest scaffolds; a horizontal red line in (A) shows the total assembled bases in the new genome and a vertical dashed line in (A) and (B) is positioned after the 14th largest scaffold."<br><br>8) L437 "caused by the presence haplotigs" -> typo "of haplotigs"<br><br>We have corrected this typo in the main text.<br><br>L458-463: "This suggests that the higher number of predicted genes in the Hawaiian Pocillopora species is not caused by the presence of haplotigs in the genome assembly, although this likely contributes to the slightly higher number of duplicated BUSCO genes in the Hawaiian Poc. acuta, or by the presence of fragmented genes models, because the number of fragmented BUSCO genes and the gene statistics suggest that the majority are full length."
**Additional Information:**	
**Question**	**Response**
Are you submitting this manuscript to a special series or article collection?	No
**Experimental design and statistics**<br><br>Full details of the experimental design and statistical methods used should be given in the Methods section, as detailed in our Minimum Standards Reporting Checklist. Information essential to interpreting the data presented should be made available in the figure legends.<br><br>Have you included all the information requested in your manuscript?	Yes
**Resources**	Yes

Powered by Editorial Manager® and ProduXion Manager® from Aries Systems Corporation

A description of all resources used, including antibodies, cell lines, animals and software tools, with enough information to allow them to be uniquely identified, should be included in the Methods section. Authors are strongly encouraged to cite Research Resource Identifiers (RRIDs) for antibodies, model organisms and tools, where possible.<br><br>Have you included the information requested as detailed in our Minimum Standards Reporting Checklist?	
**Availability of data and materials**<br><br>All datasets and code on which the conclusions of the paper rely must be either included in your submission or deposited in publicly available repositories (where available and ethically appropriate), referencing such data using a unique identifier in the references and in the “Availability of Data and Materials” section of your manuscript.<br><br>Have you have met the above requirement as detailed in our Minimum Standards Reporting Checklist?	Yes

Powered by Editorial Manager® and ProduXion Manager® from Aries Systems Corporation

Manuscript Click here to access/download;Manuscript;Hawaiian_coral_genomes_R1_v3-

High-quality genome assembles from key Hawaiian coral species

Timothy G. Stephens (ts942@sebs.rutgers.edu)[1,*], JunMo Lee (leejunmo331@gmail.com)[2], YuJin Jeong (lpple0826@knu.ac.kr)[2], Hwan Su Yoon (hsyoon2011@skku.edu)[3], Hollie M. Putnam (hputnam@uri.edu)[4], Eva Majerová (majerova@hawaii.edu)[5], and Debashish Bhattacharya (dbhattac@rutgers.edu)[1]

[1]Department of Biochemistry and Microbiology, Rutgers University, New Brunswick, NJ 08901, USA.
[2]Department of Oceanography, Kyungpook National University, Daegu, Buk-gu 41566, Korea.
[3]Department of Biological Sciences, Sungkyunkwan University, Suwon 16419, Korea.
[4]Department of Biological Sciences, University of Rhode Island; Kingston, RI 02881, USA.
[5]Hawai‘i Institute of Marine Biology, PO Box 1346 Kāne‘ohe HI 96744, USA.

*Corresponding author (ts942@sebs.rutgers.edu)

Timothy Gordon Stephens [0000-0003-1554-7175];
JunMo Lee [0000-0002-5610-543X];
YuJin Jeong [0000-0003-3097-7747];
Hwan Su Yoon [0000-0001-9507-0105];
Hollie M Putnam [0000-0003-2322-3269];
Eva Majerová [0000-0001-7815-7890];
Debashish Bhattacharya [0000-0003-0611-1273]

Abstract

Background

Coral reefs house about 25% of marine biodiversity and are critical for the livelihood of many communities by providing food, tourism revenue, and protection from wave surge. These magnificent ecosystems are under existential threat from anthropogenic climate change. Whereas extensive ecological and physiological studies have addressed coral response to environmental stress, high-quality reference genome data are lacking for many of these species. The latter issue hinders efforts to understand the genetic basis of stress resistance and to design informed coral conservation strategies.

Results

We report genome assemblies from four key Hawaiian coral species, *Montipora capitata*, *Pocillopora acuta*, *Pocillopora meandrina*, and *Porites compressa*. These species, or members of these genera, are distributed worldwide and therefore of broad scientific and ecological importance. For *M. capitata*, an initial assembly was generated from short-read Illumina and long-read PacBio data, which was then scaffolded into 14 putative chromosomes using Omni-C sequencing. For *Poc. acuta*, *Poc. meandrina,* and *Por. compressa,* high-quality assemblies were generated using short-read Illumina and long-read PacBio data. The *Poc. acuta* assembly is from a triploid individual, making it the first reference genome of a non-diploid coral animal.

Conclusions

These assemblies are significant improvements over available data and provide invaluable resources for supporting multi-omics studies into coral biology, not just in Hawai‘i, but also in other regions, where related species exist. The *Poc. acuta* assembly provides a platform for studying polyploidy in corals and its role in genome evolution and stress adaptation in these organisms.

Keywords

Coral; Scleractinia; *Montipora capitata*; *Pocillopora acuta*; *Pocillopora meandrina*; *Porites compressa*; chromosome-level genome assembly; ploidy; triploid

Background

Montipora capitata (NCBI:txid46704, marinespecies.org:taxname:287697), *Pocillopora acuta* (NCBI:txid1491507, marinespecies.org:taxname:759099), *Pocillopora meandrina* (NCBI:txid46732, marinespecies.org:taxname:206964), and *Porites compressa* (NCBI:txid46720, marinespecies.org:taxname:207236) are species of scleractinian corals that are widespread in the Hawaiian Islands, with *M. capitata* and *Por. compressa* being dominant reef builders. These species are members of cosmopolitan genera, with closely related taxa inhabiting reefs across the Great Barrier Reef and the Coral Triangle [1-3], as well as other regions, such as *Pocillopora* in Panama [4]. In recent years, due to their critical importance to Hawaiian reef ecosystems and the growing risks posed by climate change, these four species have become the subject of many stress (including thermal [5-7] and acidification [8, 9]), microbiome [10, 11], and population genomic [12-15] studies (among many others). Given this heightened interest, there is a pressing need to generate high-quality reference genome data from Hawaiian species to empower future research.

A genome assembly for *M. capitata* was published in 2019 by our group [16] using Pacific Biosciences (PacBio) RSII data. This assembly was significantly larger (886 Mbp) than other coral genomes available at that time (ca. 300-500 Mbp), and is larger than any *Montipora* species genome [17, 18] that has since been published. This initial assembly contains a high number (>18% [19]) of duplicated BUSCO genes, suggesting the presence of haplotigs (i.e., sequences derived from different homologous chromosomes) that were not removed during the assembly process. There are currently published genomes for three *Pocillopora* [4, 20, 21] species, none of which are from Hawaiʻi. One of these is a *Poc. acuta* isolate collected from Lombok, Indonesia [22] that was generated using Illumina short-read data. This genome assembly is highly fragmented, consisting of 168,465 scaffolds, and whereas it does have a scaffold N50 of 147 Kbp, the contig N50 is only 9,649 bp. The completeness of the genes predicted in this genome is not high, with only 56% of the core eukaryotic genes [20] identified in the reported "*ab initio*" predicted gene set. A second set of predicted genes inferred using RNA-seq evidence (termed the "experimental" set) contains 93% of core eukaryotic genes, however, this set does not have predicted open reading frames (i.e., it includes both coding and non-coding genes), making it difficult to make a direct comparison with other published genomes. There are currently three

Porites species with published genomes [23-25] which are of high completeness and reasonable contiguity, however, none are from Hawai‘i.

As the cost of genome sequencing, in particular, long-read methods continues to decrease, opportunities arise to generate genome data from understudied species or species that have genomes of lower quality that would benefit from the improvement gained from newer technologies. Furthermore, methods such as Dovetail Omni-C, which provides long range linkage information, enables the generation of genome assemblies that are at (or near) chromosomal-level resolution. In this study, we generated an improved reference genome assembly for our previously published Hawaiian *M. capitata* using long-read PacBio, short-read Illumina, and newly generated Omni-C data, that is of chromosome-level resolution. The 14 largest scaffolds resulting from this assembly likely represent the 14 chromosomes predicted in *Montipora* species [26]. We also generated, using PacBio HiFi data (i.e., circular consensus corrected PacBio reads), high-quality genome assemblies for two *Pocillopora* and one *Porites* species. The *Poc. acuta* isolate is a triploid, making it the first non-diploid coral genome to be sequenced.

Data description

Sample collection and processing

The four coral species targeted in this study were collected from Kāne‘ohe Bay, Hawai‘i. For *M. capitata*, the initial PacBio and Illumina-based assembly was generated using sperm DNA (see [16]). Input DNA for the Dovetail Genomics approach, using the Omni-C assay and workflow, was a bleached nubbin (a ~5 x 5cm fragment) from a colony that was greatly reduced in algal symbionts (GPS coordinates: 21.474465, -157.834468; SRA BioSample: SAMN21845729). This fragment was collected under Hawai‘i Department of Aquatic Resources Special Activity Permit 2019-60, snap frozen in liquid nitrogen, and stored at -80°C before it was shipped on dry ice to Dovetail Genomics for processing using their Omni-C assay and workflow.

For *Poc. meandrina*, one nubbin (a ~5 x 5cm fragment) was collected from an adult colony from Reef 13 (GPS coordinates: 21.450803, -157.794692) on 2020-09-05 (SRA BioSample: SAMN21845732, SAMN21845733, and SAMN21845734) under DAR-2021-33, Amendment

No. 1 to HIMB. This nubbin was selected for DNA extraction as it was bleached and would have a greatly reduced algal symbiont density. High molecular weight DNA was extracted using the QIAGEN Genomic-tip 100/G (Cat #: 10223), the QIAGEN Genomic DNA Buffer Set (Cat #: 19060), QIAGEN RNase A (100mg/mL concentration: Cat #: 19101), QIAGEN Proteinase K (Cat #: 19131), and DNA lo-bind tubes (Eppendorf Cat #: 022431021). Briefly, a clipping of the coral fragment was placed in a cleaned and sterilized mortar and pestle and ground to powder on liquid nitrogen. High molecular weight DNA was then extracted according to the manufacturer's instructions for preparation of tissue samples in the QIAGEN Genomic DNA Handbook (version 06/2015).

For *Poc. acuta*, one nubbin was collected from an adult colony from a reef next to the Hawai‘i Institute of Marine Biology (GPS coordinates: 21.436056, -157.786861) on 2018-09-05 (SRA BioSample: SAMN22898959) under Special Activity Permit 2019-60. This nubbin was selected for DNA extraction as it was bleached and would have a greatly algal reduced symbiont density. High molecular weight DNA was extracted using the QIAGEN Genomic-tip 100/G approach outlined for *Poc. meandrina* above. High molecular weight DNA from *Poc. meandrina* and *Poc. acuta* was sent to DNA Link Sequencing Lab for sequencing on their PacBio Sequel 2 (PacBio Sequel II System, RRID:SCR_017990) and Illumina NovaSeq 6000 platforms (Illumina NovaSeq 6000 Sequencing System, RRID:SCR_020150).

For *Por. compressa*, DNA was extracted from sperm released at 11 pm on 09 June 2017 from a single colony in Kāne‘ohe Bay, O‘ahu. Total genomic DNA was extracted using the CTAB protocol and the DNeasy Blood and Tissue Kit (Qiagen, Germany) with subsequent clean-up steps. Genomic data were generated using the PacBio RS II platform (PacBio RS II Sequencing System, RRID:SCR_017988). To increase the sequence quality of the assembly, a polishing step was done using the Arrow consensus caller. To this end, we generated a total of 20 Gbp of high-throughput sequencing data (Illumina HiSeq2000; 100 bp paired-end library) as follows. The whole-genome sequencing library of *Por. compressa* was prepared using the Truseq Nano DNA Prep Kit (550bp) protocol following the manufacturer's instructions. Randomly sheared genomic DNA was ligated with index adapters and purified. The ligated products were size-selected for

300-400 bp and amplified using the adapter-specific primers. Library quality was checked using a 2100 BioAnalyzer (Agilent Technologies, Santa Clara, CA, USA).

RNA Extractions

RNA was extracted by clipping a small piece of coral using clippers sterilized in 10% bleach, deionized water, isopropanol, and RNAse free water, and then placed in a 2 mL Fisherbrand™ Pre-Filled Bead Mill microcentrifuge tube containing 0.5mm glass beads (Fisher Scientific Catalog. No 15-340-152) with 1000 µL of Zymo DNA/RNA shield. A two-step extraction protocol was used to extract RNA and DNA, with the first step as a "soft" homogenization to reduce shearing of RNA or DNA. Tubes were vortexed at high speed for 1 and 2 minutes for *Poc. acuta* and *M. capitata* fragments, respectively. The supernatant was removed and designated as the "soft extraction". Second, an additional 500 µL of Zymo DNA/RNA shield was added to the bead tubes and placed in a Qiagen TissueLyser for 1 minute at 20 Hz. The supernatant was removed and designated as the "hard extraction". Subsequently, 300 µL of sample from both soft and hard homogenate was extracted with the Zymo Quick-DNA/RNA Miniprep Plus Kit (Zymo Cat D7003) Protocol with the following modifications. RNA quantity (ng_µL) was measured with a ThermoFisher Qubit Fluorometer, DNA quality was assessed using gel electrophoresis, and RNA quality was measured with an Agilent TapeStation System.

Haploid genome assembly of Hawaiian coral species

A diagram depicting the genome assembly, gene prediction, and functional annotation workflow used for each of the Hawaiian coral species is presented in Figure 1. The long-read genome sequencing data (PacBio) from the Hawaiian coral species were initially assembled using CANU (Canu, RRID:SCR_015880) (v2.2; default options) [27]. The PacBio reads from *M. capitata* (78.3 Gbp; Supplementary Table S1) and *Por. compressa* (63.3 Gbp) were generated using the PacBio RSII platform (giving the '-pacbio' parameter to the CANU assembler). The PacBio reads for *Poc. meandrina* (311.8 Gbp; Supplementary Table S1), and *Poc. acuta* (239.1 Gbp) were generated using the PacBio HiFi platform (giving the '-pacbio-hifi' parameter to the CANU assembler). An error correction step (nucleotide correction of assembly) using the initial assemblies of *M. capitata* (1.2 Gbp; Supplementary Table S2), *Por. compressa* (1.0 Gbp), *Poc. meandrina* (0.7 Gbp), and *Poc. acuta* (1.1 Gbp) was done using bowtie2 (Bowtie 2,

RRID:SCR_016368) v2.4.2 [31] and the Pilon program (Pilon, RRID:SCR_014731) v1.23 [28] with the Illumina short-read sequencing data (27.4 Gbp for *M. capitata*; 20.9 Gbp for *Por. compressa*; 27.2 Gbp for *Poc. meandrina*, and 23.0 Gbp for *Poc. acuta*; Supplementary Table S1). Before using the Illumina data, quality trimming and adapter clipping of the raw reads were done using Trimmomatic (Trimmomatic, RRID:SCR_011848) v0.39 [29]. To remove potential contaminant sequences, assembly results were analyzed using BLASTn (BLASTN, RRID:SCR_001598) (*e*-value cutoff = $1e^{-10}$) analysis with the nr database (downloaded: Feb. 2019). To estimate genome size and ploidy of the Hawaiian coral species, *k*-mer analysis was done using Jellyfish (21-mer) [30] with the Illumina short-read data.

An additional step was performed to identify any scaffolds in the coral genome assemblies that are putatively derived from the algal (Symbiodiniaceae) symbionts. Each of the four assemblies was compared against a custom database of all published Symbiodiniaceae genomes [23, 31-35] (Supplementary Table S3) using BLASTn (v2.10.1; -max_target_seqs 2000). The resulting BLAST hits were filtered, retaining only those with an *e*-value < $1e^{-20}$ and a bitscore > 1000. Hits to the *Cladocopium* sp. C15 genome [23] were also removed because this assembly is from a holobiont sequencing project (i.e., was assembled from a metagenome sample) and is, therefore, more likely to be contaminated with coral sequences than the other Symbiodiniaceae data that were derived from unialgal cultures. Overlapping filtered BLAST hits were merged and their coverage of each coral scaffold was calculated using bedtools (v2.29.2) [36]. The regions covered by merged BLAST hits on scaffolds with >10% and >1% of their bases covered by BLASTn hits were extracted and compared against the NCBI nt database using the online BLASTn tool (default settings; accessed 21 July 2022). All of the regions on scaffolds with >10% and >1% hit coverage had similarity to coral rRNA sequences in the NCBI nt database (Supplementary Table S4), suggesting that their similarity to Symbiodiniaceae genomes does not represent contamination. Therefore, no additional scaffolds were removed from the coral genome assemblies.

To reconstruct haploid genomes using the initial assemblies of the Hawaiian coral species, we used the following protocol. First, we predicted repetitive DNA sequences in the initial assemblies and constructed soft-masked assemblies. Repetitive DNA elements were identified using the RepeatModeler pipeline (RepeatModeler, RRID:SCR_015027) v2.0. [37-39] which includes RECON (RECON, RRID:SCR_021170) v1.08 and RepeatScout (RepeatScout,

RRID:SCR_014653) v1.0.6 as *de novo* repeat finding programs. We used the default options for l-mer size and removed low-complexity and tandem repeats. To classify repeat content, the libraries were constructed from giri repbase (Repbase, RRID:SCR_021169). The consensus sequences of repeat families were used to analyze corresponding repeat regions with RepeatMasker (RepeatMasker, RRID:SCR_012954) v4.1.1. The second step in the protocol was to infer assemblies as haploid genomes using the HaploMerger2 (HM2) program (the latest release, 20180603) [40] and the soft-masked assemblies. The third step was validation of duplicated eukaryotic core genes in the haploid genome assemblies using the Benchmarking Universal Single-Copy Orthologs ((BUSCO, RRID:SCR_015008)) program (v4.1.4; genome-based analysis with eukaryota_odb10 dataset) [41]. The final step was to repeat the HM2 analysis until the number of duplicated eukaryotic core genes decreased to under 1%, or the value could not be decreased any further in the haploid assemblies (Supplementary Table S2). The purged assembly of *M. capitata* was sent to Dovetail Genomics along with an additional coral fragment (see above) that was used for high molecular weight DNA extraction for analysis using their Omni-C assay and HiRise v2.2.0 assembly workflow. A total of 56.5 million read-pairs of Dovetail Genomics Omni-C sequencing data (Supplementary Table S1) were generated and used for scaffolding. This step produced a final genome assembly that was at putative chromosome level resolution for *M. capitata*.

Gene prediction and functional annotation

Quality trimming and adapter removal from the RNA sequencing (RNA-seq) data in the Hawaiian coral species (77.5 Gbp for *M. capitata*, 76.5 Gbp for *Por. compressa*, 656.7 Gbp for *Poc. acuta*, and 10.6 Gbp for *Poc. meandrina*; Supplementary Table S1) were done using Trimmomatic (v0.39; default options) [29]. These data were assembled using Trinity (Trinity, RRID:SCR_013048) v2.11 with the default option of *de novo* transcriptome assembly [42, 43]. The trimmed RNA-seq raw reads and the assembled transcriptomes were aligned to the haploid genome assemblies using the STAR (STAR, RRID:SCR_004463) aligner (v2.6.0c; default options for the raw reads) and the STARlong aligner (v2.6.0c; --runMode alignReads --alignIntronMin 10 --seedPerReadNmax 100000 --seedPerWindowNmax 1000 --alignTranscriptsPerReadNmax 100000 --alignTranscriptsPerWindowNmax 10000), respectively [44]. Based on each alignment (i.e., bam file), gene predictions were done using the BRAKER2

pipeline v2.1.5 [45], which includes GeneMark-ET [46] and AUGUSTUS (Augustus, RRID:SCR_008417) [47] with default (automatically optimized) options. When the gene models predicted in the same region of the genome by the two gene prediction approaches (i.e., RNA-seq and assembled transcript-based BRAKER gene models) differed, the best (e.g., longest non-chimeric) gene model was manually selected, based on the results of a web-BLASTp search (*e*-value cutoff = $1.e^{-5}$ cutoff). Functional annotation of gene models was done using the NCBI Conserved Domain Search (CD-Search) [48], the eggNOG-mapper [49], and the KEGG Automatic Annotation Server (KAAS) [50].

Genomes of corals used for comparative analysis

The genome assemblies and predicted genes from the four *Montipora* (*M. cactus* [17], *M. capitata* from the Hawaiian Waiopae tide pools [18], *M. efflorescens* [17], and the previous version of the Hawaiian *M. capitata* isolate [16] that we assembled in this study), three *Pocillopora* (*Poc. damicornis* [4], *Poc. acuta* [from Indonesia] [22], and *Poc. verrucosa* [21]), and four *Porites* (*Por. astreoides* [25], *Por. australiensis* [24], *Por. lutea* [23], and *Por. rus* [51]) species were retrieved from their respective repositories (Supplementary Table S5) and used for comparative analysis with the assemblies generated in this study. The *M. cactus* and *M. efflorescens* genome assemblies [17] were filtered, retaining only scaffolds identified by Yuki, Go [19] as not being haplotigs. The updated gene models from Yuki, Go [19] were used in place of those available with the original assemblies. For species where just the gene modes were provided (in gff format), gffread v0.11.6 (-S -x cdsfile -y pepfile) [52] was used to infer the protein and CDS sequences. Open Reading Frames (ORFs) were predicted in the RNA-Seq based “experimental” genes predicted in the Indonesian *Poc. acuta* isolate [22], using TransDecoder (TransDecoder, RRID:SCR_017647) v5.5.0. HMMER (Hmmer, RRID:SCR_005305) v3.1b2 was used to query the candidate ORFs against the Pfam (Pfam, RRID:SCR_004726) database (release 33.1; i-Evalue < 0.001) and BLASTp (BLASTP, RRID:SCR_001010) (v2.10.1; -max_target_seqs 1 -evalue 1e-5) was used to search candidate ORFs against the SwissProt database (release 2020_05), with the resulting homology information used by TransDecoder (TransDecoder, RRID:SCR_017647) to guide ORF prediction. Only the longest transcript per gene had ORFs predicted and single-exon genes

without strand information were assumed to be from the forward/positive strand (TransDecoder will change the strand of single exon genes if required, based on the results of ORF prediction).

Genome size estimation

The genome size and ploidy of the new (this study) and published *Montipora*, *Pocillopora,* and *Porites* species (except the Indonesian *Poc. acuta* which does not have read data available to download, *Por. rus* which only had reads from the holobiont [i.e., reads from the coral, algal symbiont, and associated bacteria] available, and *Por. astreoides* which only had PacBio long reads available) were estimated using the GenomeScope2 and Smudgeplot tools [53]. For each species, the available short-read genome sequencing data were retrieved from NCBI SRA (Supplementary Table S5), trimmed using cutadapt (cutadapt, RRID:SCR_011841) v3.5 [54] (-q 20 --minimum-length 25 -a AGATCGGAAGAGCACACGTCTGAACTCCAGTCA -A AGATCGGAAGAGCGTCGTGTAGGGAAAGAGTGT), and decomposed into *k*-mers using Jellyfish [30] (v2.3.0; k=21). The *k*-mer frequency histogram produced by Jellyfish (using the 'jellyfish histo' command) was imported into GenomeScope2 with a theoretical diploid model fitted with the data (Fig. 2C, D, and F and Supplementary Fig. S1); a theoretical triploid model was fitted with the Hawaiian *Poc. acuta* data (Fig. 2E and Supplementary Fig. S1F) because it was found to be a triploid after initial analysis using Smudgeplot and GenomeScope2. Smudgeplot was run using the *k*-mers extracted by Jellyfish (Jellyfish, RRID:SCR_005491), with thresholds for the lower *k*-mer coverage cutoff (just after the minimum between the initial error peak and the first major peak) and upper *k*-mer coverage cutoff (8.5 times the coverage of the first major coverage peak) chosen for each species using the GenomeScope2 profile shown in Supplementary Figure S1. The "smudge plots" shown in Supplementary Figure S1 were generated using the haploid coverage values estimated by GenomeScope2. The cutoffs used when running Smudgeplot for each species are shown in Supplementary Table S5.

Confirmation of sample ploidy

The program nQuire [55] (retrieved 7/7/2021), which uses the frequency distribution of bi-allelic variant sites inferred from aligned reads to model the ploidy of a sample, was used to verify the ploidy of the four genomes sequenced in this study. Briefly, bowtie2 (Bowtie 2, RRID:SCR_016368) v2.4.4 ('--very-sensitive --no-unal') was used to align the trimmed (by

cutadapt; described previously) Illumina short-reads against their respective genome assemblies; aligned reads were coordinate sorted using samtools (SAMTOOLS, RRID:SCR_002105) v1.11 [56]. The aligned and sorted BAM files were converted into "BIN" files using nQuire ('nQuire create -q 20 -c 20 -x'), filtering for reads with a minimum mapping quality of 20 and sites with a minimum coverage of 20. Denoised BIN files were created using the "nQuire denoise" command run on the initial BIN files. The delta Log-Likelihood values for each ploidy model (diploid, triploid, and tetraploid) was calculated by the "nQuire lrdmodel" command for each of the initial and denoised BIN files. The lower the delta Log-Likelihood value of a given model the better fit it is for the frequency distribution of the bi-allelic variant sites extracted from the aligned reads; the ploidy of the sample is there for assumed to be the ploidy model with the lowest delta Log-Likelihood value. The nQuire results are shown in Supplementary Table S6.

Assessment of completeness using BUSCO

The "completeness" of the genome assemblies and predicted genes (published in this study and from previous studies; Supplementary Table S7) were assessed using BUSCO v5.0.0 ('--mode genome' and '--mode protein', respectively) with the eukaryota_odb10 (release 2020-09-10) and metazoa_odb10 datasets (release 2021-02-24) [57].

Analysis of extra-chromosomal scaffolds

The proteins predicted on the extra-chromosomal scaffolds (i.e., the scaffolds that do not comprise the 14 putative chromosomes) in the *M. capitata* assembly were compared against the proteins from the chromosomal scaffolds using BLASTp v1.10.1 [58]; the resulting hits were filtered using an *e*-value cutoff < $1x10^{-5}$. Additional filtering steps were applied to produce two sets of hits: for the first (lenient) set, hits were retained if they had a query coverage of > 75% and an identity > 75%, with the single best (*e*-value-based) top hit kept for each query sequence; for the second (stringent) set, hits were retained if they had a query coverage of > 95% and an identity > 95%, with the single best (*e*-value-based) top hit kept for each query sequence. The lenient filtered top hits were used to determine if the extra-chromosomal scaffolds tend to encode genes that have similarity to a single, or multiple, chromosomes. For this analysis, only proteins with top hits to the chromosomal scaffolds (i.e., proteins with hits that have an *e*-value < $1x10^{-5}$,

query coverage > 75%, and an identity > 75%) were considered, and only scaffolds with multiple proteins with top hits were considered.

Data Validation and Quality Control

Montipora capitata genome assemblies

The *M. capitata* assembly generated in the study (assembly version V3.0; hereinafter the "new" Hawaiian *M. capitata* genome assembly) has fewer assembled bases (781 Mbp vs. 886 Mbp) and scaffolds (1,699 vs. 3,043), and a vastly improved N50 (47.7 Mbp vs. 0.54 Mbp; Supplementary Table S7), compared to the assembly of the same Hawaiian *M. capitata* isolate (hereinafter the "old" Hawaiian *M. capitata* genome assembly) that was previously published by our group [16]. The 14 largest scaffolds in the new assembly, ranging in size from ~22 to ~69 Mbp, likely represent the 14 chromosomes predicted in other *Montipora* species (Figs. 2A and B) [26]. These putative chromosomes total 680 Mbp of assembled sequence, which is only slightly larger than the estimated genome size of 644 Mbp (Fig. 2C; estimated by GenomeScope2 [53] using *k*-mers of size 21 bp). The estimated genome size of the other published *Montipora* species is ~700 Mbp, whereas the estimated genome size of the new Hawaiian *M. capitata* genome is 644 Mbp (although the assembly is a little larger; see discussion below). This suggests that species in the genus *Montipora* have genomes that are marginally smaller than 700 Mbp in size.

The *M. capitata* isolate that was sequenced appears to be a diploid, with a good fit between its *k*-mer frequency histogram and the theoretical diploid model implemented in GenomeScope2 (black line in Fig. 2C and Supplementary Fig. S1A), and a clear "smudge" (bright yellow region in Supplementary Fig. S1A) of *k*-mer pairs with a coverage of 2n and a normalized coverage of 1/2; all of which suggests that the sample is diploid. nQuire also predicted that the *M. capitata* sample was a diploid (i.e., the diploid model had the lowest delta Log-Likelihood value; Supplementary Table S6), supporting the results of GenomeScope2 and Smudegeplot.

Compared with the old assembly, the new *M. capitata* assembly has a slightly higher BUSCO completeness for both the Metazoa (from 95.2% to 95.7%, respectively) and Eukaryota (from 97.7% to 99.2%, respectively) datasets but a significantly reduced number of duplicated BUSCO genes for both the Metazoa (from 21.2% to 1.6%, respectively) and Eukaryota (from 22.0% to 1.2%, respectively) datasets (Fig. 3A and 3B; Supplementary Table S7). The high

number of duplicated BUSCO genes in the old assembly is likely a result of haplotigs that were not removed during the assembly process; this problem appears to have been resolved in the new assembly. Compared with the other published *Montipora* genomes, the new *M. capitata* assembly is the most contiguous and complete to date, with a significantly higher N50 (47.7 Mbp compared to the next best of 1.2 Mbp in *M. efflorescens*) and BUSCO completeness (e.g., 99.2% Eukaryota dataset completeness compared to the next best of 92.1% in *M. cactus*). Because the same PacBio and Illumina libraries were used to construct the new and old assemblies, the significant improvement observed in the new assembly is attributed to the use of a different hybrid assembly approach, combined with the Dovetail Omni-C library preparation and scaffolding with the HiRise (v2.2.0) software.

***Pocillopora* genome assemblies**

The *Poc. acuta* genome assembly generated in this study (hereinafter the "Hawaiian *Poc. acuta*") is larger (408 Mbp) than *Poc. acuta* from Indonesia (352 Mbp) [22] (Supplementary Table S7) and its estimated genome size of 353 Mbp (Fig. 2E). The size of the *Poc. meandrina* genome assembly generated in this study (377 Mbp) is comparable to that in the published Indonesian *Poc. acuta* (352 Mbp) [22] and *Poc. verrucosa* (381 Mbp) [21] species, but is larger than in *Poc. damicornis* (234 Mbp) [4] (Supplementary Table S7). Although the latter is likely under-assembled given its smaller size relative to the estimated genome size for that species. Moreover, the estimated genome sizes for these species appears to be around 330-350 Mbp, with the assemblies being 350-380 Mbp in size (excluding the Hawaiian *Poc. acuta* [see discussion below]). This suggests that species in the genus *Pocillopora* have genomes that are ~350 Mbp in size.

The Hawaiian *Poc. acuta* isolate that was sequenced is a triploid; the presence of three major peaks in the *k*-mer frequency histogram (at ~17x, ~35, and ~51x) which fit the triploid model implemented by GenomeScope2 (black line Fig. 2E and Supplementary Fig. S1F), and the clear "smudge" (bright yellow region in Supplementary Fig. S1F) of *k*-mer pairs with a coverage of ~3n and a normalized coverage of 1/3, all suggests that the sample is triploid. nQuire also predicts that the *Poc. acuta* is a triploid (Supplementary Table S6), supporting the results of GenomeScope2 and Smudegeplot. For *Poc. meandrina*, GenomeScope2 (Fig. 2D), Smudgeplot

(Supplementary Fig. S1E), and nQuire (Supplementary Table S6) all predict that the isolate that was sequenced is a diploid.

The BUSCO completeness of the Hawaiian *Poc. acuta* genome is improved for both the Metazoa (96.1%), and Eukaryota (98.5%) datasets compared to the Indonesian *Poc. acuta* assembly (89.4% and 91.4%, respectively) and the other *Pocillopora* assemblies (~91-95% and 91-98%, respectively; Supplementary Table S7 and Fig. 3A and 3B). However, the Hawaiian assembly does have a slightly higher proportion of duplicated BUSCO genes (2.5% and 2.0% in the Metazoa and Eukaryota datasets) compared with some (the Indonesian *Poc. acuta* and *Poc. damicornis* genomes which have <1% in both datasets) but not all (the *Poc. verrucosa* genome which has 2.9% and 5.5%, respectively) of the published genomes. This is likely a result of the Hawaiian *Poc. acuta* being a triploid; haplotig removal programs (i.e., HaploMerger2 [40]) are generally designed for use with diploid species, therefore, it is unsurprising that they were unable to fully resolve the assembly given the added complexity associated with resolving assemblies of higher ploidy genomes. Regardless, the Hawaiian *Poc. acuta* assembly is more contiguous (i.e., higher N50 and fewer scaffolds) then the other *Pocillopora* genomes and is the first assembly generated from a non-diploid coral. The *Poc. meandrina* genome has a BUSCO completeness (96.1% for the Metazoa and 98.8% for the Eukaryota datasets) that is just as high as the Hawaiian *Poc. acuta* genome, but with fewer duplicated BUSCO genes (1.2% and 0.4%, respectively), suggesting that this assembly has minimal retained haplotigs (Supplementary Table S7 and Fig. 3A and 3B).

***Porites compressa* genome assembly**

The size of the *Por. compressa* genome assembly generated in this study (593 Mbp) is similar to the published *Por. australiensis* (576 Mbp) [24] and *Por. lutea* (552 Mbp) [23] genomes, and a little smaller than *Por. astreoides* (677 Mbp). The estimated genome sizes for these species appears to be around 525-550 Mbp (excluding *Por. astreoides*, *Por. lutea* and *Por. rus*), with the assemblies coming in at around 550-600 Mbp. The high number of duplicated BUSCO genes in the *Por. astreoides* assembly (11.5% and 14.9% for the Metazoa and Eukaryota datasets, respectively; Supplementary Table S7 and Fig. 3A and 3B) suggests that its larger assembly size (compared with the other *Porites* species) is likely explained by retained haplotigs. The genome assembly (470 Mbp) and estimated genome size (405 Mbp) of *Por. rus* is smaller than the other

Porites isolates however, these data were generated from holobiont samples (i.e., samples with both coral, algal symbiont, and associated bacteria DNA present) using a metagenomic binning strategy. The difference in this approach compared with how the other *Porites* genomes were processed likely explain the difference between the sizes. *Por. lutea* has an estimated genome size of 694 Mbp, which is significantly larger than the other *Porites* species and its assembled genome. Whereas this suggests that the *Por. lutea* genome is under-assembled (comprising only ~80% of the estimated genome) its relatively high completeness (95.3% and 98.5% for the Metazoa and Eukaryota datasets, respectively) suggests that the genome size has been overestimated, possibly driven by sequencing error or other factors associated with sample preparation or collection from the field. These results indicate that species in the genus *Porites* have genomes that are just under 600 Mbp in size. For *Por. compressa*, GenomeScope2 (Fig. 2F), Smudgeplot (Supplementary Fig. S1I), and nQuire (Supplementary Table S6) all predict that the isolate sequenced is a diploid.

The BUSCO completeness of the *Por. compressa* assembly is slightly higher (95.5% for the Metazoa and 99.2% for the Eukaryota datasets) compared to the *Por. astreoides* (93.2% and 98.0%, respectively), *Por. australiensis* (91.6% and 94.9%, respectively), *Por. lutea* (95.3% and 98.5%, respectively), and *Por. rus* (69.6% and 67.1%, respectively) assemblies (Supplementary Table S7 and Fig. 3A and 3B), but has a much higher N50 (4 Mbp) compared to the published species (0.41, 0.55, 0.66, and 0.14 Mbp, respectively) and fewer scaffolds (608 vs. 3,051, 4,983, 2,975, and 14,982, respectively). The published genome assemblies also have many more gaps (~0-29% of assembled bases are 'N' characters) compared to *Por. compressa* (0%), demonstrating that the new assembly is of equally high completeness compared to the published species, but with a much higher contiguity.

Predicted protein-coding genes

For *M. capitata*, 54,384 protein-coding genes were predicted in the new assembly compared with 63,227 predicted in the old version (Supplementary Table S7). In the new assembly, 56.68% of the predicted protein-coding genes were assigned putative functions using CD-Search, 44.26% using eggNOG-mapper, and 21.20% using KAAS (Supplementary Table S8). The reduction in the number of predicted genes in the new *M. capitata* assembly, compared with the published version, is likely driven by its reduced assembly size, with many of the missing genes likely

arising from haplotigs retained in the old assembly, that were removed in the new version. The BUSCO completeness of the predicted genes is improved in the new assembly (95.2% of the Metazoa and 96.5% for the Eukaryota BUSCO datasets; Fig. 3C and 3D) compared with the old assembly (94.0% and 93.3%, respectively), and the number of duplicated BUSCO genes is reduced in the new assembly (2.3% and 1.2%, respectively) compared to the published (18.2% and 18.8%, respectively). The predicted gene set from the new Hawaiian *M. capitata* assembly also has > 4.2% and > 3.5% more complete BUSCO genes (from the Metazoa and Eukaryota datasets, respectively) recovered compared to the other published isolates, demonstrating that the gene models predicted in the new assembly are also highly complete. Whereas increase in the number of genes predicted in the new Hawaiian *M. capitata* genome, compared with the published species, could be attributed to differences in the workflows used to predicted the genes in these species [31], it is also likely driven by the higher completeness and contiguity of the new genome assembly.

There are 33,730 predicted protein-coding genes in the Hawaiian *Poc. acuta* and 31,840 in the *Poc. meandrina* genome assemblies, which is ~4,000–8,000 more than predicted in other *Pocillopora* species (Supplementary Table S7). In *Poc. acuta*, 67.76% of the predicted protein-coding genes were assigned putative functions using CD-Search, 49.76% using eggNOG-mapper, and 32.35% using KAAS, and in *Poc. meandrina*, 69.44% of the predicted protein-coding genes were assigned putative functions using CD-Search, 51.76% using eggNOG-mapper, and 33.66% using KAAS (Supplementary Table S8). The number of complete BUSCO genes from the Metazoa and Eukaryota BUSCO datasets is > 6% higher in the new Hawaiian *Poc. acuta* and *Poc. meandrina* species then in the other *Pocillopora* species; the Hawaiian *Poc. acuta* also has 29.6% and 31.3% (respectively) more complete BUSCO genes recovered than the Indonesian *Poc. acuta* (Supplementary Table S7; Fig. 3C and 3D). The number of duplicated BUSCO genes is > 0.7% and > 2.3% (respectively) higher in the Hawaiian *Poc. acuta* gene set compared with the published *Pocillopora* species however, this was expected given the increased size of the genome assembly. The proportion of fragmented BUSCO genes is > 0.9% and > 2% lower (Metazoa and Eukaryota BUSCO datasets, respectively) lower in the Hawaiian *Pocillopora* species compared with the published species. The average transcript length and the number of CDSs per transcript of the Hawaiian *Pocillopora* genes (~1,350 bp and ~6.6, respectively) are congruent with the predicted genes of the published *Pocillopora* species

(~1,100–1,900 bp and ~5.5-7.5, respectively). This suggests that the higher number of predicted genes in the Hawaiian *Pocillopora* species is not caused by the presence of haplotigs in the genome assembly, although this likely contributes to the slightly higher number of duplicated BUSCO genes in the Hawaiian *Poc. acuta*, or by the presence of fragmented genes models, because the number of fragmented BUSCO genes and the gene statistics suggest that the majority are full length. Therefore, the higher number of predicted genes in this species can be (at least partially) attributed to the more complete and contiguous genome assemblies of the Hawaiian *Pocillopora* species relative to published species.

There are 44,130 predicted protein-coding genes in the Hawaiian *Por. compressa* genome assembly (Supplementary Table S7), which is > 8,000 more genes than predicted in the *Por. australiensis* (35,910) and *Por. lutea* (31,126) genomes, 4,677 more than in the *Por. rus* (39,453) genome, and 20,506 less than in the *Por. astreoides* (64,636) genome. In *Por. compressa*, 63.91% of the predicted protein-coding genes were assigned putative functions using CD-Search, 46.22% using eggNOG-mapper, and 27.48% using KAAS (Supplementary Table S8). The number of complete BUSCO genes from the Metazoa and Eukaryota BUSCO datasets is > 4% higher in *Por. compressa* than in the published *Porites* species (Supplementary Table S7; Fig. 3C and 3D). The number of duplicated BUSCO genes in *Por. compressa* is similar to *Por. lutea* and *Por. rus* but lower than in *Por. astreoides* and *Por. australiensis*, and the number of fragmented BUSCO genes in *Por. compressa* is much lower (> 1.9% and > 5.1%, respectively) than in the published species. As with the previous Hawaiian genomes, we attribute the higher number of predicted genes in this species to a more complete and contiguous assembly, relative to the published data.

Similarity between *Montipora capitata* chromosomal and extra-chromosomal scaffolds

There are 1,685 scaffolds (totaling ~101 Mbp) in the new *M. capitata* assembly that were not placed into the 14 putative chromosomes by the scaffolding software. Given that the size of the 14 chromosomal sequences totals ~680 Mbp, which is close to the estimated genome size of 644 Mbp, it is possible that the extra-chromosomal sequences represent retained haplotigs. To explore this issue, we compared the predicted genes in the extra-chromosomal (6,545 protein-coding genes) and chromosomal (47,839) scaffolds to determine how similar the protein content is between the two sets of scaffolds and to see if the extra-chromosomal proteins tend to be

contained within a single chromosome, suggesting that they are likely to be retained haplotigs. Out of the 6,546 proteins encoded in the extra-chromosomal scaffolds, 3,896 (59.53%) have hits to chromosomal proteins with > 75% query coverage and > 75% identity, and 1,623 (24.80%) have hits to chromosomal proteins with > 95% query coverage and > 95% identity. This suggests that whereas the two sets of scaffolds encode many similar (although not identical) proteins, the protein inventory of the extra chromosomal scaffolds only partially overlaps with the gene inventory of the chromosomal scaffolds (we would expect them to have a high level of overlap if they were haplotigs). Furthermore, the extra-chromosomal scaffolds encode 12% of the total predicted genes but, when analyzed separately using BUSCO, have only 1.9% of the Metazoa and 1.6% of the Eukaryota BUSCO genes recovered. This conflict between the number of predicted genes in the scaffolds and the number of BUSCO genes suggests that these scaffolds cannot be easily explained as unresolved haplotigs. Finally, of the 3,896 proteins with top hits in the leniently filtered dataset (hit with > 75% query coverage and > 75% identity), 2,748 (70.53%) were on scaffolds with other proteins with top hits to different chromosomes. This suggests that the extra-chromosomal scaffolds have significant structural differences when compared to the chromosomes. These results suggest that the extra-chromosomal scaffolds do not comprise retained haplotigs however, given their significant size, which increases the assembly size well above the estimated size, additional analyses will need to be done to determine the placement of these sequences in the chromosomes and the genes they encode.

Potential implications

The substantial improvement in the contiguity and completeness of the assemblies and predicted genes from the Hawaiian *M. capitata*, *Poc. meandrina*, *Poc. acuta*, and *Por. compressa* species will enable many follow-up studies. The chromosome-level assembly of the *M. capitata* isolate will not only serve as a key reference genome for future population studies focusing on this species in Hawaii, but it will also enable more detailed studies on genome content (such as repeats), gene content, and gene synteny with other species from reefs across the world. The *Poc. acuta* genome, although not at chromosome-level resolution, is the most complete available for this genus and will be a valuable model for not only comparative analysis, but for analysis of ploidy in corals. As the first assembly ever generated from a non-diploid coral, this data will open up new questions surrounding the role of ploidy in coral evolution and adaptation and how

this phenomenon is involved in the lifecycle of this species and potentially other *Pocillopora* species, both in Hawai‘i and other reefs across the world. These questions are critical, because an understanding of how changes in ploidy evolve in these corals, particularly in response to stress, will help us model the response of these ecosystems to anthropogenic climate change, and may even provide a new avenue of research for the development of stress resistant "super" corals.

Data availability

The SRA Run IDs of the Omni-C data generated from the Hawaiian *M. capitata*, the PacBio and Illumina genome data used for genome assembly, and the RNA-seq data used for gene prediction are listed in Supplementary Table S1 for each species. The genome assemblies, predicted genes, and functional annotations for the Hawaiian *M. capitata* is available at Rutgers's website [59], for *Poc. acuta* at Rutgers's website [60], *Poc. meandrina* at Rutgers's website [61], *Por. compressa* at Rutgers's website [62]. The data from the other *Montipora*, *Pocillopora*, and *Porites* species used in this study are available from their respective repositories listed in Supplementary Table S5. Supporting data and materials are available in the GigaDB database [63], with individual datasets for *M. capitata* [64], *P. acuta* [65], *P. meandrina* [66] and *P. compressa* [67].

Additional Files

Supplementary Figure S1. GenomeScope2 (left) and Smudgeplot (right) profiles for (**A**) Hawaiian *M. capitata* (this study), (**B**) Waiopae tide pools *M. capitata*, (**C**) *M. cactus*, (**D**) *M. efflorescens*, (**E**) *Poc. meandrina* (this study), (**F**) Hawaiian *Poc. acuta* (this study), (**G**) Indonesian *Poc. acuta*, (**H**) *Poc. verrucose*, (**I**) *Por. compressa* (this study), (**J**) *Por. australiensis*, and (**K**) *Por. lutea*. The profiles were computed for each species using 21-mers generated from the trimmed short-read data listed in Supplementary Table S5.

Abbreviations

bp: base pairs

BUSCO: Benchmarking Universal Single-Copy Orthologs

Gbp: gigabase pairs

HM2: HaploMerger2

Kbp: Kilobase pairs
Mbp: megabase pairs
NCBI: National Center for Biotechnology Information
PacBio: Pacific BioSciences
SRA: Sequencing Read Archive

Conflict of Interests

The authors declare that they have no other competing interests.

Funding

This work was supported by the National Science Foundation grant NSF-OCE 1756616, the Catalyst Science Fund grant 2020-008, the National Institute of Food and Agriculture and United States Department of Agriculture Hatch grant NJ01180, and the National Aeronautics and Space Administration grant 80NSSC19K0462 awarded to DB. DB and HSY were also supported by the Collaborative Genome Program of the Korea Institute of Marine Science and Technology Promotion (KIMST) funded by the Ministry of Oceans and Fisheries (MOF) (20180430). JML was supported by research grants from the National Research Foundation of Korea [NRF, 2020R1C1C1010193 (JML)] and 'The project to make multi-ministerial national biological research resources more advanced' program through the Korea Environment Industry & Technology Institute (KEITI) funded by the Korea Ministry of Environment [MOE (2021003420004)]. HMP was supported by the USDA National Institute of Food and Agriculture, Hatch Formula project accession number 1017848 and the National Science Foundation grant NSF-OCE 1756623. EM was supported by the Paul G. Allen Family Foundation.

Author contributions

DB conceived the project with HMP and JML. TGS, JML, and YJJ did the bioinformatic analyses, HSY provided sequencing resources, and HMP led the coral sample collection and processing with EM. TGS wrote the manuscript draft with JML, and all authors commented on and approved the submitted version.

Acknowledgements

We acknowledge support from the research and administrative staff at the Hawai‘i Institute of Marine Biology where some of this work was done.

References

1. van Oppen MJH, Koolmees EM and Veron JEN. Patterns of evolution in the scleractinian coral genus *Montipora* (Acroporidae). Marine Biology. 2004;144 1:9-18. doi:10.1007/s00227-003-1188-3.
2. Forsman ZH, Concepcion GT, Haverkort RD, Shaw RW, Maragos JE and Toonen RJ. Ecomorph or endangered coral? DNA and microstructure reveal hawaiian species complexes: *Montipora dilatata/flabellata/turgescens* & *M. patula/verrilli*. PLoS One. 2010;5 12:e15021. doi:10.1371/journal.pone.0015021.
3. Schmidt-Roach S, Miller KJ, Lundgren P and Andreakis N. With eyes wide open: A revision of species within and closely related to the *Pocillopora damicornis* species complex (Scleractinia; Pocilloporidae) using morphology and genetics. Zoological Journal of the Linnean Society. 2014;170 1:1-33. doi:doi.org/10.1111/zoj.12092.
4. Cunning R, Bay RA, Gillette P, Baker AC and Traylor-Knowles N. Comparative analysis of the *Pocillopora damicornis* genome highlights role of immune system in coral evolution. Sci Rep. 2018;8 1:16134. doi:10.1038/s41598-018-34459-8.
5. Williams A, Pathmanathan JS, Stephens TG, Su X, Chiles EN, Conetta D, et al. Multi-omic characterization of the thermal stress phenome in the stony coral *Montipora capitata*. PeerJ. 2021;9:e12335. doi:10.7717/peerj.12335.
6. Mayfield AB, Chen YJ, Lu CY and Chen CS. The proteomic response of the reef coral *Pocillopora acuta* to experimentally elevated temperatures. PLoS One. 2018;13 1:e0192001. doi:10.1371/journal.pone.0192001.
7. Henley EM, Quinn M, Bouwmeester J, Daly J, Zuchowicz N, Lager C, et al. Reproductive plasticity of Hawaiian *Montipora* corals following thermal stress. Sci Rep. 2021;11 1:12525. doi:10.1038/s41598-021-91030-8.
8. Putnam HM, Davidson JM and Gates RD. Ocean acidification influences host DNA methylation and phenotypic plasticity in environmentally susceptible corals. Evol Appl. 2016;9 9:1165-78. doi:10.1111/eva.12408.
9. Jury CP, Delano MN and Toonen RJ. High heritability of coral calcification rates and evolutionary potential under ocean acidification. Sci Rep. 2019;9 1:20419. doi:10.1038/s41598-019-56313-1.
10. Padilla-Gamino JL, Pochon X, Bird C, Concepcion GT and Gates RD. From parent to gamete: vertical transmission of *Symbiodinium* (Dinophyceae) ITS2 sequence assemblages in the reef building coral *Montipora capitata*. PLoS One. 2012;7 6:e38440. doi:10.1371/journal.pone.0038440.
11. Damjanovic K, Menendez P, Blackall LL and van Oppen MJH. Mixed-mode bacterial transmission in the common brooding coral *Pocillopora acuta*. Environ Microbiol. 2020;22 1:397-412. doi:10.1111/1462-2920.14856.

12. Cunha RL, Forsman ZH, Belderok R, Knapp ISS, Castilho R and Toonen RJ. Rare coral under the genomic microscope: timing and relationships among Hawaiian *Montipora*. BMC Evol Biol. 2019;19 1:153. doi:10.1186/s12862-019-1476-2.
13. Johnston EC, Forsman ZH, Flot JF, Schmidt-Roach S, Pinzon JH, Knapp ISS, et al. A genomic glance through the fog of plasticity and diversification in *Pocillopora*. Sci Rep. 2017;7 1:5991. doi:10.1038/s41598-017-06085-3.
14. Aurelle D, Pratlong M, Oury N, Haguenauer A, Gélin P, Magalon H, et al. Population genomics of *Pocillopora* corals: insights from RAD-sequencing. 2021-10-12 2021.
15. Caruso C, de Souza MR, Ruiz-Jones L, Conetta D, Hancock J, Hobbs C, et al. Genetic patterns in *Montipora capitata* across an environmental mosaic in Kāne'ohe Bay. bioRxiv. 2021:2021.10.07.463582. doi:10.1101/2021.10.07.463582.
16. Shumaker A, Putnam HM, Qiu H, Price DC, Zelzion E, Harel A, et al. Genome analysis of the rice coral *Montipora capitata*. Sci Rep. 2019;9 1:2571. doi:10.1038/s41598-019-39274-3.
17. Shinzato C, Khalturin K, Inoue J, Zayasu Y, Kanda M, Kawamitsu M, et al. Eighteen coral genomes reveal the evolutionary origin of *Acropora* strategies to accommodate environmental changes. Molecular Biology and Evolution. 2021;38 1:16-30. doi:10.1093/molbev/msaa216.
18. Helmkampf M, Bellinger MR, Geib S, Sim SB and Takabayashi M. Draft genome of the rice coral *Montipora capitata* obtained from linked-read sequencing. Genome Biol Evol. 2019;11 7:2045-54. doi:10.1093/gbe/evz135.
19. Yuki Y, Go S, Yuna Z, Hiroshi Y and Chuya S. Comparative genomics highlight the importance of lineage-specific gene families in evolutionary divergence of the coral genus, *Montipora*. BMC Ecology and Evolution. 2021; doi:10.21203/rs.3.rs-944849/v1.
20. Parra G, Bradnam K and Korf I. CEGMA: A pipeline to accurately annotate core genes in eukaryotic genomes. Bioinformatics. 2007;23 9:1061-7. doi:10.1093/bioinformatics/btm071.
21. Buitrago-Lopez C, Mariappan KG, Cardenas A, Gegner HM and Voolstra CR. The genome of the cauliflower coral *Pocillopora verrucosa*. Genome Biol Evol. 2020;12 10:1911-7. doi:10.1093/gbe/evaa184.
22. Vidal-Dupiol J, Chaparro C, Pratlong M, Pontarotti P, Grunau C and Mitta G. Sequencing, *de novo* assembly and annotation of the genome of the scleractinian coral, *Pocillopora acuta*. bioRxiv. 2020:698688. doi:10.1101/698688.
23. Robbins SJ, Singleton CM, Chan CX, Messer LF, Geers AU, Ying H, et al. A genomic view of the reef-building coral *Porites lutea* and its microbial symbionts. Nat Microbiol. 2019;4 12:2090-100. doi:10.1038/s41564-019-0532-4.
24. Shinzato C, Takeuchi T, Yoshioka Y, Tada I, Kanda M, Broussard C, et al. Whole-genome sequencing highlights conservative genomic strategies of a stress-tolerant, long-lived scleractinian coral, *Porites australiensis* Vaughan, 1918. Genome Biol Evol. 2021;13 12 doi:10.1093/gbe/evab270.
25. Wong KH and Putnam HM. The genome of the mustard hill coral, *Porites astreoides*. GIGAbyte. 2022; doi:10.46471/gigabyte.65.
26. Kenyon JC. Models of reticulate evolution in the coral genus *Acropora* based on chromosome numbers: Parallels with plants. Evolution. 1997;51 3:756-67. doi:10.1111/j.1558-5646.1997.tb03659.x.

27. Nurk S, Walenz BP, Rhie A, Vollger MR, Logsdon GA, Grothe R, et al. HiCanu: accurate assembly of segmental duplications, satellites, and allelic variants from high-fidelity long reads. Genome Res. 2020;30 9:1291-305. doi:10.1101/gr.263566.120.
28. Walker BJ, Abeel T, Shea T, Priest M, Abouelliel A, Sakthikumar S, et al. Pilon: an integrated tool for comprehensive microbial variant detection and genome assembly improvement. PLoS One. 2014;9 11:e112963. doi:10.1371/journal.pone.0112963.
29. Bolger AM, Lohse M and Usadel B. Trimmomatic: A flexible trimmer for Illumina sequence data. Bioinformatics. 2014;30 15:2114-20. doi:10.1093/bioinformatics/btu170.
30. Marcais G and Kingsford C. A fast, lock-free approach for efficient parallel counting of occurrences of *k*-mers. Bioinformatics. 2011;27 6:764-70. doi:10.1093/bioinformatics/btr011.
31. Chen YB, Gonzalez-Pech RA, Stephens TG, Bhattacharya D and Chan CX. Evidence that inconsistent gene prediction can mislead analysis of dinoflagellate genomes. J Phycol. 2020;56 1:6-10. doi:10.1111/jpy.12947.
32. Dougan KE, Bellantuono AJ, Kahlke T, Abbriano RM, Chen Y, Shah S, et al. Whole-genome duplication in an algal symbiont serendipitously confers thermal tolerance to corals. bioRxiv. 2022:2022.04.10.487810. doi:10.1101/2022.04.10.487810.
33. Li T, Yu L, Song B, Song Y, Li L, Lin X, et al. Genome improvement and core gene set refinement of *Fugacium kawagutii*. Microorganisms. 2020;8 1 doi:10.3390/microorganisms8010102.
34. González-Pech RA, Stephens TG, Chen Y, Mohamed AR, Cheng Y, Shah S, et al. Comparison of 15 dinoflagellate genomes reveals extensive sequence and structural divergence in family Symbiodiniaceae and genus *Symbiodinium*. BMC Biology. 2021;19 1:73. doi:10.1186/s12915-021-00994-6.
35. Nand A, Zhan Y, Salazar OR, Aranda M, Voolstra CR and Dekker J. Genetic and spatial organization of the unusual chromosomes of the dinoflagellate *Symbiodinium microadriaticum*. Nat Genet. 2021;53 5:618-29. doi:10.1038/s41588-021-00841-y.
36. Quinlan AR and Hall IM. BEDTools: A flexible suite of utilities for comparing genomic features. Bioinformatics. 2010;26 6:841-2. doi:10.1093/bioinformatics/btq033.
37. Benson G. Tandem repeats finder: a program to analyze DNA sequences. Nucleic Acids Res. 1999;27 2:573-80. doi:10.1093/nar/27.2.573.
38. Bao Z and Eddy SR. Automated de novo identification of repeat sequence families in sequenced genomes. Genome Res. 2002;12 8:1269-76. doi:10.1101/gr.88502.
39. Price AL, Jones NC and Pevzner PA. De novo identification of repeat families in large genomes. Bioinformatics. 2005;21 Suppl 1:i351-8. doi:10.1093/bioinformatics/bti1018.
40. Huang S, Kang M and Xu A. HaploMerger2: Rebuilding both haploid sub-assemblies from high-heterozygosity diploid genome assembly. Bioinformatics. 2017;33 16:2577-9. doi:10.1093/bioinformatics/btx220.
41. Simao FA, Waterhouse RM, Ioannidis P, Kriventseva EV and Zdobnov EM. BUSCO: assessing genome assembly and annotation completeness with single-copy orthologs. Bioinformatics. 2015;31 19:3210-2. doi:10.1093/bioinformatics/btv351.
42. Haas BJ, Papanicolaou A, Yassour M, Grabherr M, Blood PD, Bowden J, et al. *De novo* transcript sequence reconstruction from RNA-seq using the Trinity platform for reference generation and analysis. Nat Protoc. 2013;8 8:1494-512. doi:10.1038/nprot.2013.084.

43. Grabherr MG, Haas BJ, Yassour M, Levin JZ, Thompson DA, Amit I, et al. Full-length transcriptome assembly from RNA-Seq data without a reference genome. Nat Biotechnol. 2011;29 7:644-52. doi:10.1038/nbt.1883.
44. Dobin A, Davis CA, Schlesinger F, Drenkow J, Zaleski C, Jha S, et al. STAR: Ultrafast universal RNA-seq aligner. Bioinformatics. 2013;29 1:15-21. doi:10.1093/bioinformatics/bts635.
45. Bruna T, Hoff KJ, Lomsadze A, Stanke M and Borodovsky M. BRAKER2: Automatic eukaryotic genome annotation with GeneMark-EP+ and AUGUSTUS supported by a protein database. NAR Genom Bioinform. 2021;3 1:lqaa108. doi:10.1093/nargab/lqaa108.
46. Lomsadze A, Burns PD and Borodovsky M. Integration of mapped RNA-Seq reads into automatic training of eukaryotic gene finding algorithm. Nucleic Acids Res. 2014;42 15:e119. doi:10.1093/nar/gku557.
47. Stanke M, Keller O, Gunduz I, Hayes A, Waack S and Morgenstern B. AUGUSTUS: *Ab initio* prediction of alternative transcripts. Nucleic Acids Res. 2006;34 Web Server issue:W435-9. doi:10.1093/nar/gkl200.
48. Marchler-Bauer A, Bo Y, Han L, He J, Lanczycki CJ, Lu S, et al. CDD/SPARCLE: functional classification of proteins via subfamily domain architectures. Nucleic Acids Res. 2017;45 D1:D200-D3. doi:10.1093/nar/gkw1129.
49. Huerta-Cepas J, Forslund K, Coelho LP, Szklarczyk D, Jensen LJ, von Mering C, et al. Fast Genome-Wide Functional Annotation through Orthology Assignment by eggNOG-Mapper. Molecular Biology and Evolution. 2017;34 8:2115-22. doi:10.1093/molbev/msx148.
50. Moriya Y, Itoh M, Okuda S, Yoshizawa AC and Kanehisa M. KAAS: an automatic genome annotation and pathway reconstruction server. Nucleic Acids Research. 2007;35:W182-W5. doi:10.1093/nar/gkm321.
51. Celis JS, Wibberg D, Ramirez-Portilla C, Rupp O, Sczyrba A, Winkler A, et al. Binning enables efficient host genome reconstruction in cnidarian holobionts. Gigascience. 2018;7 7 doi:10.1093/gigascience/giy075.
52. Pertea G and Pertea M. GFF Utilities: GffRead and GffCompare [version 1; peer review: 2 approved]. F1000Research. 2020;9 304 doi:10.12688/f1000research.23297.1.
53. Ranallo-Benavidez TR, Jaron KS and Schatz MC. GenomeScope 2.0 and Smudgeplot for reference-free profiling of polyploid genomes. Nat Commun. 2020;11 1:1432. doi:10.1038/s41467-020-14998-3.
54. Martin M. Cutadapt removes adapter sequences from high-throughput sequencing reads. EMBnetjournal. 2011;17 1:3. doi:10.14806/ej.17.1.200.
55. Weiss CL, Pais M, Cano LM, Kamoun S and Burbano HA. nQuire: A statistical framework for ploidy estimation using next generation sequencing. BMC Bioinformatics. 2018;19 1:122. doi:10.1186/s12859-018-2128-z.
56. Danecek P, Bonfield JK, Liddle J, Marshall J, Ohan V, Pollard MO, et al. Twelve years of SAMtools and BCFtools. Gigascience. 2021;10 2 doi:10.1093/gigascience/giab008.
57. Manni M, Berkeley MR, Seppey M, Simão FA and Zdobnov EM. BUSCO Update: Novel and streamlined workflows along with broader and deeper phylogenetic coverage for scoring of eukaryotic, prokaryotic, and viral genomes. Molecular Biology and Evolution. 2021; doi:10.1093/molbev/msab199.

58. Camacho C, Coulouris G, Avagyan V, Ma N, Papadopoulos J, Bealer K, et al. BLAST+: Architecture and applications. BMC Bioinformatics. 2009;10:421. doi:10.1186/1471-2105-10-421.

59. Genome data for Montipora capitata from http://cyanophora.rutgers.edu/montipora/ (Version 3).

60. Genome data for Pocillopora acuta from http://cyanophora.rutgers.edu/Pocillopora_acuta/ (Version 2).

61. Genome data for Pocillopora meandrina from http://cyanophora.rutgers.edu/Pocillopora_meandrina/ (Version 1).

62. Genome data for Porites compressa from http://cyanophora.rutgers.edu/Porites_compressa/ (Version 1).

63. Stephens TG, Lee J, Jeong Y, Yoon HS, Putnam HM, Majerová E, et al. Supporting data for "High-quality genome assemblies from key Hawaiian coral species" GigaScience Database. 2022. http://dx.doi.org/10.5524/102259.

64. Stephens TG, Lee J, Jeong Y, Yoon HS, Putnam HM, Majerová E, et al. Chromosome-level genome assembly of Montipora capitata GigaScience Database. 2022. http://dx.doi.org/10.5524/102268

65. Stephens TG, Lee J, Jeong Y, Yoon HS, Putnam HM, Majerová E, et al. Genome assembly of a triploid Pocillopora acuta GigaScience Database. 2022. http://dx.doi.org/10.5524/102269

66. Stephens TG, Lee J, Jeong Y, Yoon HS, Putnam HM, Majerová E, et al. Genome assembly of Pocillopora meandrina GigaScience Database. 2022. http://dx.doi.org/10.5524/102270

67. Stephens TG, Lee J, Jeong Y, Yoon HS, Putnam HM, Majerová E, et al. Genome assembly of Porites compressa GigaScience Database. 2022. http://dx.doi.org/10.5524/102271

Tables

Table S1: Summary of read data used for genome assembly and gene prediction.

Table S2: Summary of coral assemblies before and after haplotype merging.

Table S3: List of Symbiodiniaceae genomes used to assess symbiont contamination in the coral genome assemblies.

Table S4: Top 10 BLASTn hits against the NCBI's nt database for regions of coral scaffolds with greater than a given coverage of hits to Symbiodiniaceae assembled genomes.

Table S5: Metadata for the genome and gene models downloaded for the coral species used for comparative analysis.

Table S6: Results from nQuire lrdmodel ploidy estimation for the Hawaiian coral genomes analyzed in this study.

Table S7: Comparison between the published *Montipora*, *Pocillopora*, and *Porites* genomes and those generated in this study. All statistics were calculated in this study using the available genome and gene models.

Table S8: Number of predicted protein-coding genes in each of the new Hawaiian coral genomes with functional annotations.

Figure Legends

Figure 1: Diagram depicting the genome assembly, gene prediction, and functional annotation workflow deployed in this study to assemble each of the new Hawaiian coral genomes. Programs are presented in green boxes and datasets in dark orange boxes, arrows show the flow of data through the workflow. Major input and output datasets are highlighted with bold text.

Figure 2: (**A**) Cumulative and (**B**) individual length of scaffolds in the new Hawaiian *M. capitata* genome assembly. Scaffolds were sorted by length in descending order; each point along the x-axis of (**A**) and (**B**) represents a scaffold, with the longest scaffold being the first and the shortest being the last on the x-axis of each plot. In (**A**) and (**B**) a zoomed-in section of the larger plot (indicated by a green bar along the x-axis) is shown on the right highlighting the 40 largest scaffolds; a horizontal red line in (**A**) shows the total assembled bases in the new genome and a vertical dashed line in (**A**) and (**B**) is positioned after the 14th largest scaffold. GenomeScape2 linear *k*-mer distributions of the Hawaiian (**C**) *M. capitata*, (**D**) *Poc. meandrina*, (**E**) *Poc. acuta*, and (**F**) *Por. compressa* species with theoretical diploid (or triploid for *Poc. acuta*) models shown by the black lines. The GenomeScope2 profiles were computed for each species using 21-mers generated from the trimmed short-read data listed in Supplementary Table S5.

Figure 3: Results from BUSCO analysis run using the genomes and predicted genes from all published (including this study) *Montipora*, *Pocillopora*, and *Porites* species, plus the old version of the *M. capitata* genome that our group published in 2019 [16]. BUSCO results for each species using the (**A**) Metazoa dataset (genome mode), (**B**) Eukaryota dataset (genome mode), (**C**) Metazoa dataset (protein mode), and (**D**) Eukaryota dataset (protein mode).

Figure 1

Click here to access/download;Figure;Figure_1.pdf

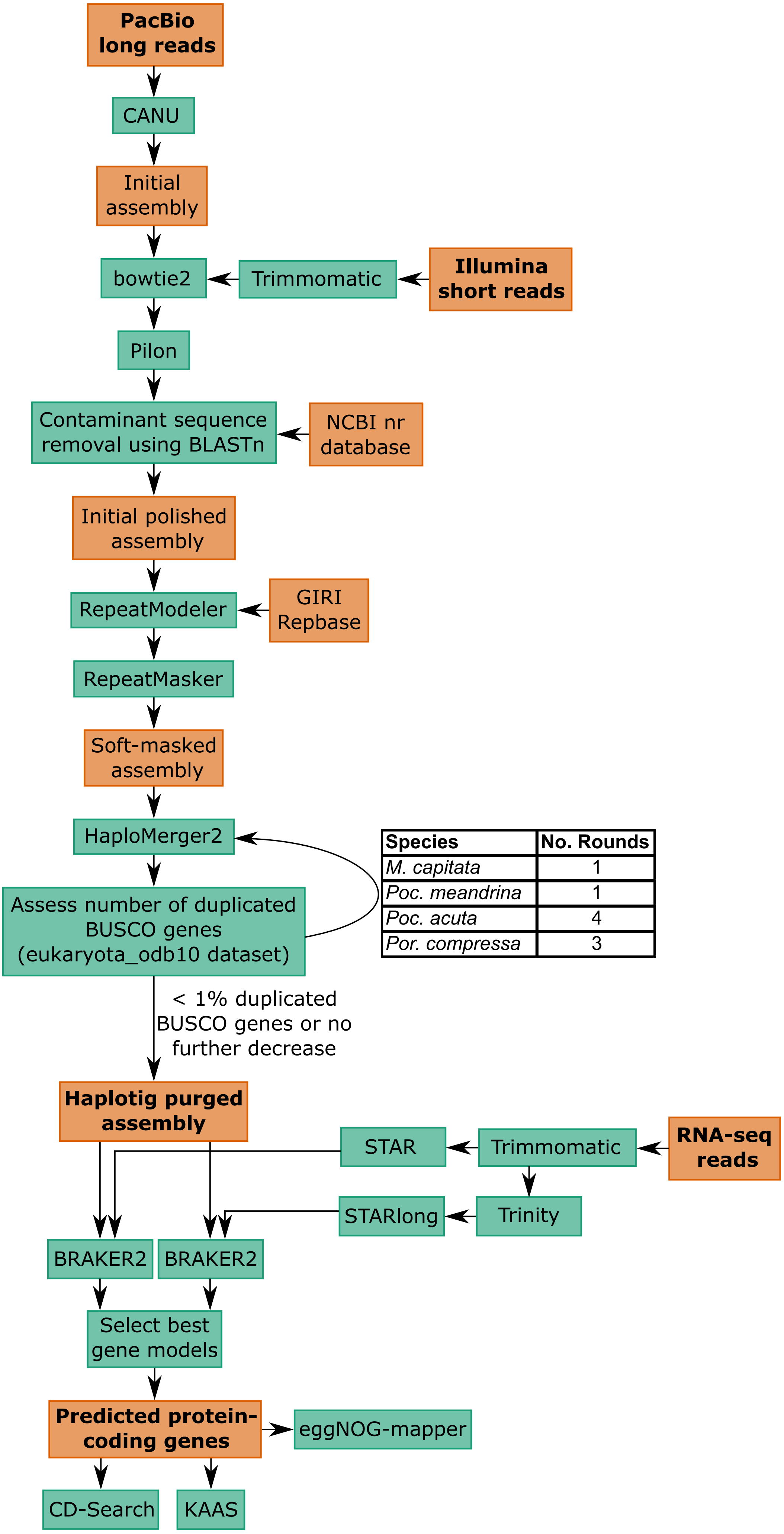

Figure 2

Click here to access/download;Figure;Figure_2.pdf

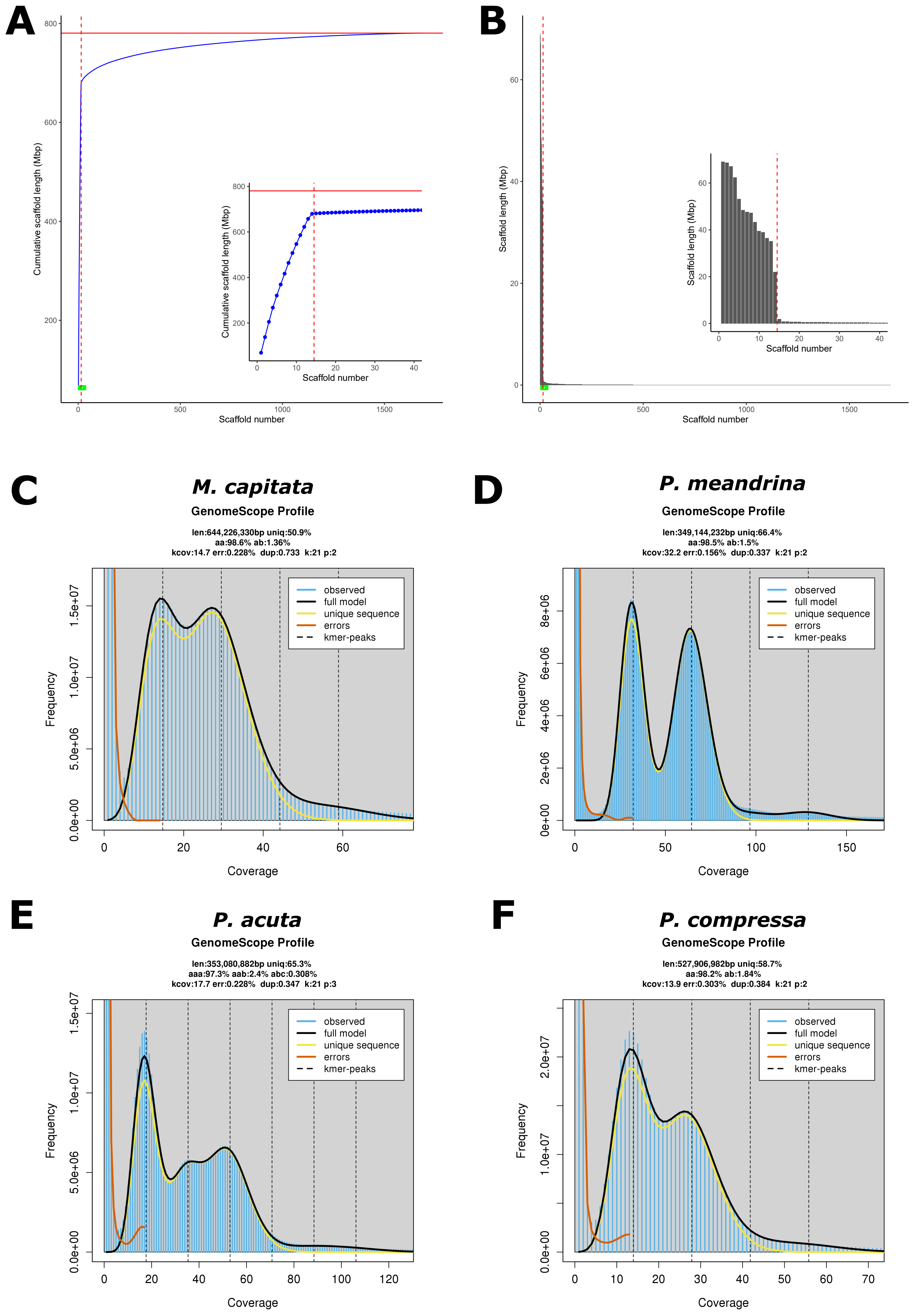

Figure 3

Click here to access/download;Figure;Figure_3.pdf

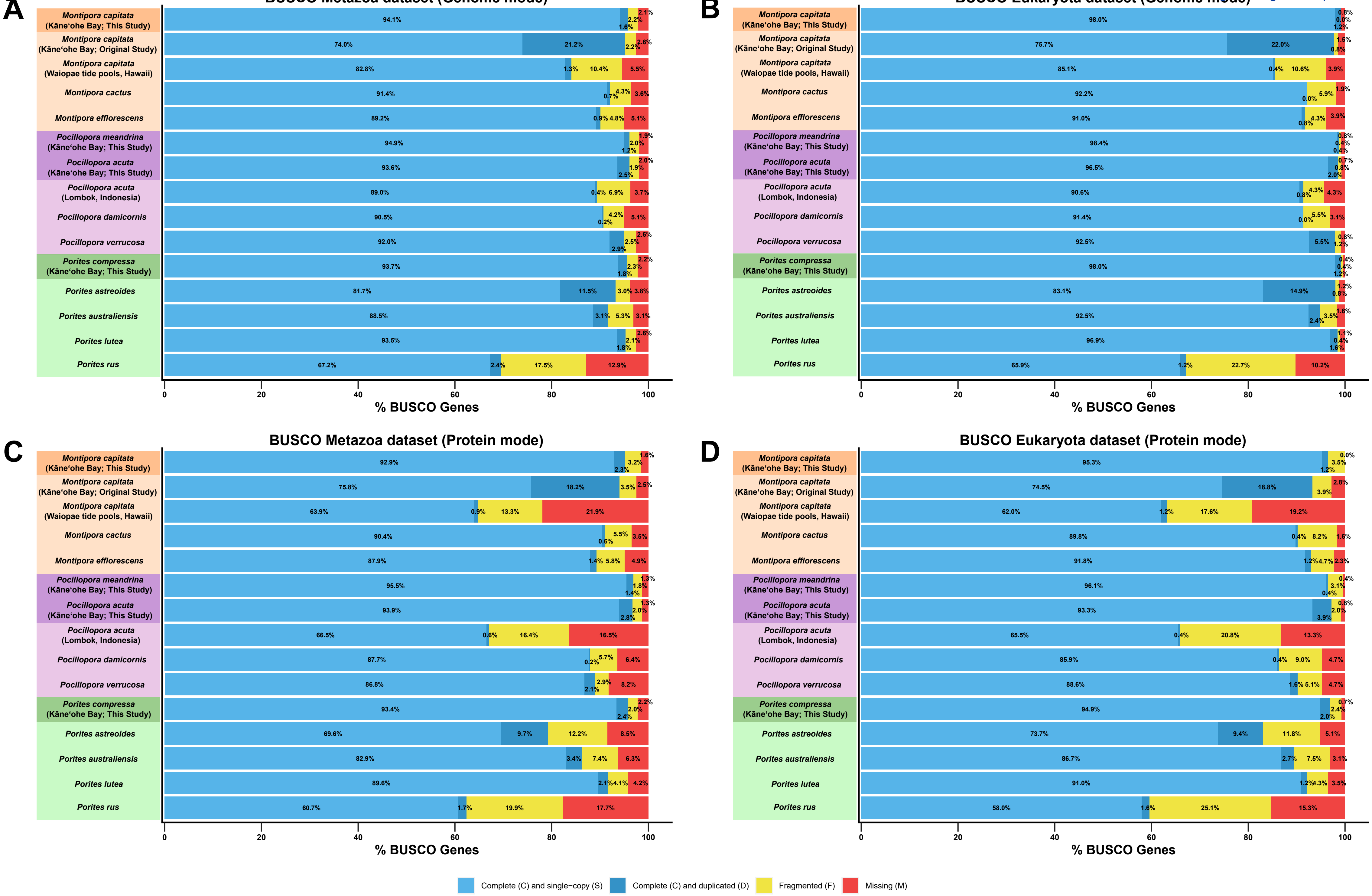

Supplementary Tables S1-S8

Click here to access/download
Supplementary Material
Supplementary_Tables.xlsx

Supplementary Figure S1

Click here to access/download
Supplementary Material
Figure_S1.pdf
