# Supplementary material for: High-quality genome assembles from key Hawaiian coral species
Source: Gigascience. 2022 Nov 9;11:giac098. doi: 10.1093/gigascience/giac098 (PMC9646523; doi:10.1093/gigascience/giac098)
Supplement: giac098_Response_to_Reviewer_Comments_Original_Submission [file giac098_response_to_reviewer_comments_original_submission.pdf]

Author's Response To Reviewer Comments

Close

Dear Editor,
We thank the two reviewers for their constructive comments on our earlier manuscript.
In this submission, we revised our manuscript based on all of these comments, and to improve readability. We have added two new supplementary tables listing the SRA Run IDs and results of functional annotation, and a figure showing our assembly and gene prediction workflow. We also added a more detailed description of the symbiont filtering approach and the results of functional annotation.

Reviewer #1: Stephens et al. reported de novo genome assemblies from four coral species in Hawaii. They constructed a chromosome-level assembly of Montipora capitata using the Omni-C sequencing technology. These genome assemblies surpass previous ones from the same species or genera in contiguity and BUSCO completeness. These genome assemblies will be helpful to the coral research community. I have a few comments for the authors to consider.

The authors would benefit from proof-read by an English editor to correct grammar and improve the manuscript's readability.

We have extensively reviewed the grammar and phrasing of the manuscript to improve its readability.

Lines 139-151, 182-191
I think it is better to summarize the information of the sequence data in tables than to describe it in the text.

We have added a new supplementary table (Table S1) listing the IDs of the SRA Runs used for genome assembly and gene prediction in this study. We have removed the lists of Run IDs from the main text and now refer to the new table where appropriate.

L144-154: "The PacBio reads from M. capitata (78.3 Gbp; Supplementary Table S1) and Por. compressa (63.3 Gbp) were generated using the PacBio RSII platform (giving the '-pacbio' parameter to the CANU assembler). The PacBio reads for Poc. meandrina (311.8 Gbp; Supplementary Table S1), and Poc. acuta (239.1 Gbp) were generated using the PacBio HiFi platform (giving the '-pacbio-hifi' parameter to the CANU assembler). An error correction step (nucleotide correction of assembly) using the initial assemblies of M. capitata (1.2 Gbp; Supplementary Table S2), Por. compressa (1.0 Gbp), Poc. meandrina (0.7 Gbp), and Poc. acuta (1.1 Gbp) was done using bowtie2 (v2.4.2; default options) [31] and the Pilon program (v1.23; default options) [28] with the Illumina short-read sequencing data (27.4 Gbp for M. capitata; 20.9 Gbp for Por. compressa; 27.2 Gbp for Poc. meandrina, and 23.0 Gbp for Poc. acuta; Supplementary Table S1)."

L202-205: "Quality trimming and adapter removal from the RNA sequencing (RNA-seq) data in the Hawaiian coral species (77.5 Gbp for M. capitata, 76.5 Gbp for Por. compressa, 656.7 Gbp for Poc. acuta, and 10.6 Gbp for Poc. meandrina; Supplementary Table S1) were done using Trimmomatic (v0.39; default options) [29]."

L527-529: "The SRA Run IDs of the Omni-C data generated from the Hawaiian M. capitata, the PacBio and Illumina genome data used for genome assembly, and the RNA-seq data used for gene prediction are listed in Supplementary Table S1 for each species."

Lines 203-205
Results of functional annotation are not described.

We had added to the manuscript additional text describing these results and a new supplementary table (Table S8) that lists the number of functionally annotated genes in each species.

L422-424: "In the new assembly, 56.68% of the predicted protein-coding genes were assigned putative

functions using CD-Search, 44.26% using eggNOG-mapper, and 21.20% using KAAS (Supplementary Table S8)."

L442-446: "In Poc. acuta, 67.76% of the predicted protein-coding genes were assigned putative functions using CD-Search, 49.76% using eggNOG-mapper, and 32.35% using KAAS, and in Poc. meandrina, 69.44% of the predicted protein-coding genes were assigned putative functions using CD-Search, 51.76% using eggNOG-mapper, and 33.66% using KAAS (Supplementary Table S8)."

L469-471: "In Por. compressa, 63.91% of the predicted protein-coding genes were assigned putative functions using CD-Search, 46.22% using eggNOG-mapper, and 27.48% using KAAS (Supplementary Table S8)."

L783-784: "Table S8: Number of predicted protein-coding genes in each of the new Hawaiian coral genomes with functional annotations."

Reviewer #2: n this work, Stephens et al present improved reference genomes from four Hawaian coral species using a combination of short and long read sequencing as well as linkage information in one assembly. They also sequence the first triploid coral. I believe this data will be a valuable resource to the larger coral community and are thus a good fit for a GigaScience Data Note. Overall, the methods are largely sound, appropriate and reproducible. Some small suggestions to improve are:

1) The manuscript would benefit from workflow diagrams describing the entire workflow and potentially a separate diagram for the assembly and annotation pipeline.

We agree with the reviewer and have added a diagram of the genome assembly, gene prediction, and functional annotation workflow to the manuscript.

L141-142: "A diagram depicting the genome assembly, gene prediction, and functional annotation workflow used for each of the Hawaiian coral species is presented in Figure 1."

L787-790: "Figure 1: Diagram depicting the genome assembly, gene prediction, and functional annotation workflow deployed in this study to assemble each of the new Hawaiian coral genomes. Programs are presented in green boxes and datasets in dark orange boxes, arrows show the flow of data through the workflow. Major input and output datasets are highlighted with bold text."

2) The improved assemblies will be beneficial to the research community. Could you clarify whether the old assemblies were utilised in any way during the construction of the improved assemblies?

We thank the Reviewer for their support of the importance of these data to the research community. The old assemblies were not used in any way during the construction of the improved assemblies. As we describe in the methods, the "long-read genome sequencing data (PacBio) of the Hawaiian coral species were initially assembled using CANU (v2.2; default options)". That is, each of the improved assemblies were constructed directly from the long and short read data and not using the existing genome assemblies as a start point. As we feel that this is adequately described in the manuscript, we have made no further changes.

3) L204: "Functional annotation of gene models was done using the NCBI Conserved Domain Search (CD-Search) [42], the eggNOG-mapper [43], and the KEGG Automatic Annotation Server (KAAS)". Is this functional data described in the manuscript? Is it available?

We will be making the results of functional annotation available through our lab website and the GigaDB data repository. We have also added to the manuscript additional text describing the functional annotation results, as well as a new supplementary table (Table S8) that lists the number of functionally annotated genes in each species.

L529-535: "The genome assemblies, predicted genes, and functional annotations for the Hawaiian M. capitata is available from http://cyanophora.rutgers.edu/montipora/ (Version 3), for Poc. acuta from http://cyanophora.rutgers.edu/Pocillopora_acuta/ (Version 2), Poc. meandrina from http://cyanophora.rutgers.edu/Pocillopora_meandrina/ (Version 1), Por. compressa from http://cyanophora.rutgers.edu/Porites_compressa/ (Version 1). The data associated with this manuscript are also available from GigaDB."

L422-424: “In the new assembly, 56.68% of the predicted protein-coding genes were assigned putative functions using CD-Search, 44.26% using eggNOG-mapper, and 21.20% using KAAS (Supplementary Table S8).”

L442-446: “In Poc. acuta, 67.76% of the predicted protein-coding genes were assigned putative functions using CD-Search, 49.76% using eggNOG-mapper, and 32.35% using KAAS, and in Poc. meandrina, 69.44% of the predicted protein-coding genes were assigned putative functions using CD-Search, 51.76% using eggNOG-mapper, and 33.66% using KAAS (Supplementary Table S8).”

L469-471: “In Por. compressa, 63.91% of the predicted protein-coding genes were assigned putative functions using CD-Search, 46.22% using eggNOG-mapper, and 27.48% using KAAS (Supplementary Table S8).”

L783-784: “Table S8: Number of predicted protein-coding genes in each of the new Hawaiian coral genomes with functional annotations.”

4) You note large differences in the number of predicted genes between species and mention assemblies qualities may impact this. Was there anything characteristic about the genes found uniquely in Por. Compress versus the other assemblies? Did you examine whether there are any functional differences between the genes?

We thank the reviewer for their insightful comment and agree that an exploration of the genes that are unique to the Por. compressa genome would make for an interesting follow-up study. We however think that such an analysis is outside the scope of a GigaScience Data Note article because it would require extensive reanalysis of the published Porites genomes (to ensure the conclusions drawn from the analysis are not the result of differences in assembly and gene prediction quality or methodology) and the exploration and discussion of the literature on Porites and coral genome evolution. We are currently performing follow-up analyses of the genomes that we are publishing in this study, plus all published coral genomes, to explore how the different forces that have shaped the genome evolution of different coral groups. As such, we believe that a rigorous analysis of the genes that are unique to the Por. compressa genome is outside the scope of a GigaScience Data Note article and we have made no additional changes to the manuscript.

5) You state "the best (longest) gene models were manually selected based on results of BLASTp search" however this is not always true. For the two methods, do you have the breakdown for the number of times the transcripts differed and if so which method predicted the longer transcript?

When gene models from the two types of gene prediction approached are visualized, using for example Geneious Prime, the differently predicted gene models are easily recognized. ‘The best (longest) gene models’ means that the “best” gene models from the two prediction approaches were selected based on a web-BLASTp search and selection of the longest non-chimeric gene models. We agree with the Reviewer that a BLASTp search will not always return the “true” gene model, however, we propose that a gene model with multiple BLASTp hits to proteins in an updated reference database should be regarded as the strongest evidence of the correct gene structure in the absence of other evidence. To select the longest non-chimeric gene models, we compared gene models (not transcripts) constructed by BRAKER using assembled transcripts or RNA-seq reads as evidence for exons. Further, both type of gene models were used because assembled transcriptome data could generally (but not always) make longer gene models, however, it can also sometimes result in chimeric gene models when UTR regions of two closely related genes overlap. There for, we used gene models from these two complementary methods, and evidence of potential chimeric gene models based on the blast results compared to reference proteins, as the basis for our selection of the “best” non-chimeric gene models. We have rephased this section of the manuscript to make this point clearer. We did not keep track of the number of differently predicted gene models or the number of times one type of prediction was correct over the other.

L213-217: “When the gene models predicted in the same region of the genome by the two gene prediction approaches (i.e., RNA-seq and assembled transcript-based BRAKER gene models) differed, the best (e.g., longest non-chimeric) gene model was manually selected, based on the results of a web-BLASTp search (e-value cutoff = 1.e-5 cutoff).”

6) Could you further explain how symbiont sequence data was handled? For one species you say "from a colony that was greatly reduced in algal symbionts" but for others no such claims are made. You speak

of general contamination filtering strategies but given this is coral you might want to specifically describe if anything specific was done for the handling of symbiont sequence.

For M. capitata, Poc. acuta, and Poc. meandrina, DNA was extracted from bleached coral nubbins, which would have reduced algal symbiont densities, and for Por. compressa, DNA was extracted from sperm, which should be free from algal symbionts. As the reviewer highlighted, this is described in the methods for M. capitata and Por. compressa but not for Poc. acuta, and Poc. meandrina. We have added these missing details to the methods section of the manuscript.

L92-93 & 104-105: “This nubbin was selected for DNA extraction as it was bleached and would have a greatly reduced algal symbiont density.”

We have added a detailed description of the symbiont sequence screening workflow to the main text of the manuscript; two additional supplementary tables were added that describe the symbiont genome assemblies used for screening and the putative functions of the coral scaffolds identified as having similarity to symbiont genomes above our chosen thresholds.

L160-176: “An additional step was performed to identify any scaffolds in the coral genome assemblies that are putatively derived from the algal (Symbiodiniaceae) symbionts. Each of the four assemblies was compared against a custom database of all published Symbiodiniaceae genomes [23, 31-35] (Supplementary Table S3) using BLASTn (v2.10.1; -max_target_seqs 2000). The resulting BLAST hits were filtered, retaining only those with an e-value < 1e-20 and a bitscore > 1000. Hits to the Cladocopium sp. C15 genome [23] were also removed because this assembly is from a holobiont sequencing project (i.e., was assembled from a metagenome sample) and is, therefore, more likely to be contaminated with coral sequences than the other Symbiodiniaceae data that were derived from unialgal cultures. Overlapping filtered BLAST hits were merged and their coverage of each coral scaffold was calculated using bedtools (v2.29.2) [36]. The regions covered by merged BLAST hits on scaffolds with >10% and >1% of their bases covered by BLASTn hits were extracted and compared against the NCBI nt database using the online BLASTn tool (default settings; accessed 21 July 2022). All of the regions on scaffolds with >10% and >1% hit coverage had similarity to coral rRNA sequences in the NCBI nt database (Supplementary Table S4), suggesting that their similarity to Symbiodiniaceae genomes does not represent contamination. Therefore, no additional scaffolds were removed from the coral genome assemblies.”

L767-771: “Table S3: List of Symbiodiniaceae genomes used to assess symbiont contamination in the coral genome assemblies.

Table S4: Top 10 BLASTn hits against the NCBI's nt database for regions of coral scaffolds with greater than a given coverage of hits to Symbiodiniaceae assembled genomes.”

7) In Figure 1A/B, it would be clearer to highlight the region blown up in the magnified images.

We agree with the Reviewer that highlighting the magnified regions would make Figure 1A and 1B (now Figure 2) clearer. We have added green bars to each of the panels to highlight the magnified regions.

L795-798: “In (A) and (B) a zoomed-in section of the larger plot (indicated by a green bar along the x-axis) is shown on the right highlighting the 40 largest scaffolds; a horizontal red line in (A) shows the total assembled bases in the new genome and a vertical dashed line in (A) and (B) is positioned after the 14th largest scaffold.”

8) L437 "caused by the presence haplotigs" -> typo "of haplotigs"

We have corrected this typo in the main text.

L458-463: “This suggests that the higher number of predicted genes in the Hawaiian Pocillopora species is not caused by the presence of haplotigs in the genome assembly, although this likely contributes to the slightly higher number of duplicated BUSCO genes in the Hawaiian Poc. acuta, or by the presence of fragmented genes models, because the number of fragmented BUSCO genes and the gene statistics suggest that the majority are full length.”

Close
