# Supplementary material for: High-quality genome assembles from key Hawaiian coral species
Source: Gigascience. 2022 Nov 9;11:giac098. doi: 10.1093/gigascience/giac098 (PMC9646523; doi:10.1093/gigascience/giac098)
Supplement: giac098_Reviewer_2_Report_Original_Submission — Matt Field, PhD -- 7/15/2022 Reviewed [file giac098_reviewer_2_report_original_submission.pdf]

Reviewer Report

Title: High-quality genome assembles from key Hawaiian coral species

Version: Original Submission **Date:** 7/15/2022

Reviewer name: Matt Field, PhD

Reviewer Comments to Author:

n this work, Stephens et al present improved reference genomes from four Hawaian coral species using a combination of short and long read sequencing as well as linkage information in one assembly. They also sequence the first triploid coral. I believe this data will be a valuable resource to the larger coral community and are thus a good fit for a GigaScience Data Note. Overall, the methods are largely sound, appropriate and reproducible. Some small suggestions to improve are:
1) The manuscript would benefit from workflow diagrams describing the entire workflow and potentially a separate diagram for the assembly and annotation pipeline.
2) The improved assemblies will be beneficial to the research community. Could you clarify whether the old assemblies were utilised in any way during the construction of the improved assemblies?
3) L204: "Functional annotation of gene models was done using the NCBI Conserved Domain Search (CD-Search) [42], the eggNOG-mapper [43], and the KEGG Automatic Annotation Server (KAAS)". Is this functional data described in the manuscript? Is it available?
4) You note large differences in the number of predicted genes between species and mention assemblies qualities may impact this. Was there anything characteristic about the genes found uniquely in Por. Compress versus the other assemblies? Did you examine whether there are any functional differences between the genes?
5) You state "the best (longest) gene models were manually selected based on results of BLASTp search" however this is not always true. For the two methods, do you have the breakdown for the number of times the transcripts differed and if so which method predicted the longer transcript?
6) Could you further explain how symbiont sequence data was handled? For one species you say "from a colony that was greatly reduced in algal symbionts" but for others no such claims are made. You speak of general contamination filtering strategies but given this is coral you might want to specifically describe if anything specific was done for the handling of symbiont sequence.
7) In Figure 1A/B, it would be clearer to highlight the region blown up in the magnified images.
8) L437 "caused by the presence haplotigs" -> typo "of haplotigs"

Level of Interest

Please indicate how interesting you found the manuscript: Choose an item.

Quality of Written English

Please indicate the quality of language in the manuscript: Choose an item.

Declaration of Competing Interests

Please complete a declaration of competing interests, considering the following questions:

- Have you in the past five years received reimbursements, fees, funding, or salary from an organisation that may in any way gain or lose financially from the publication of this manuscript, either now or in the future?
- Do you hold any stocks or shares in an organisation that may in any way gain or lose financially from the publication of this manuscript, either now or in the future?
- Do you hold or are you currently applying for any patents relating to the content of the manuscript?
- Have you received reimbursements, fees, funding, or salary from an organization that holds or has applied for patents relating to the content of the manuscript?
- Do you have any other financial competing interests?
- Do you have any non-financial competing interests in relation to this paper?

If you can answer no to all of the above, write 'I declare that I have no competing interests' below. If your reply is yes to any, please give details below.

I declare that I have no competing interests

I agree to the open peer review policy of the journal. I understand that my name will be included on my report to the authors and, if the manuscript is accepted for publication, my named report including any attachments I upload will be posted on the website along with the authors' responses. I agree for my report to be made available under an Open Access Creative Commons CC-BY license (http://creativecommons.org/licenses/by/4.0/). I understand that any comments which I do not wish to be included in my named report can be included as confidential comments to the editors, which will not be published.

Choose an item.

To further support our reviewers, we have joined with Publons, where you can gain additional credit to further highlight your hard work (see: https://publons.com/journal/530/gigascience). On publication of this paper, your review will be automatically added to Publons, you can then choose whether or not to claim your Publons credit. I understand this statement.

Yes Choose an item.
